# Polyvalent Ionic Energetic Salts Based on 4-Amino-3-hydrazino-5-methyl-1,2,4-triazole

**DOI:** 10.3390/ijms241713136

**Published:** 2023-08-24

**Authors:** Qiuhan Lin, Mingmin Zhang, Linan Zhang, Mimi Zhu, Kaiyi Qin, Pengcheng Wang

**Affiliations:** School of Chemistry and Chemical Engineering, Nanjing University of Science and Technology, Nanjing 210094, China; zmm1219@njust.edu.cn (M.Z.); zhanglinan2019@njust.edu.cn (L.Z.); 121103022194@njust.edu.cn (M.Z.); qinky0731@njust.edu.cn (K.Q.)

**Keywords:** energetic salts, triazole, methyl, thermostability, detonation performance

## Abstract

The synthesis of the new energetic material 4-amino-3-hydrazino-5-methyl-1,2,4-triazole, which shows excellent performance and reliable safety, has drawn attention recently. To fully characterize this material, a comprehensive analysis was performed using various techniques, including differential scanning calorimetry (DSC), infrared spectroscopy (IR), elemental analysis, and ^1^H and ^13^C NMR spectroscopy. Additionally, three compounds, **3**, **5** and **9**, were further characterized using single X-ray diffraction. The X-ray data suggested that extensive hydrogen bonds affect molecular structure by means of intermolecular interactions. In order to evaluate the explosive properties of these synthesized compounds, detonation pressures and velocities were calculated using EXPLO5 (V6.01). These calculations were carried out utilizing experimental data, including density and heat of formation. Among the explosives tested, compounds **7** and **8** exhibited zero oxygen balance and demonstrated exceptional detonation properties. Compound **7** achieved the highest recorded detonation pressure, at 34.2 GPa, while compound **8** displayed the highest detonation velocity, at 8887 m s^−1^.

## 1. Introduction

The synthesis and design of new energetic materials has been ongoing since the discovery of 2,4,6-trinitrotoluene (TNT) in 1863 [1]. Accompanying their growing demand in military and civilian applications, researchers have made significant progress by developing compounds such as Hexogeon (RDX) [2], Cyclotetramethylene tetranitramine (HMX) [2], and Hexanitrohexaazaisowurtzitane (CL-20) [3]. However, these explosives still face several limitations that hinder their widespread use. In recent decades, many researchers have directed their efforts towards the development of nitrogen-rich energetic materials. Because of the existence of large numbers of C-N and N-N bonds, nitrogen-rich energetic materials have a higher heat of formation [4]. Molecular structures with high nitrogen and low carbon content can enable energetic materials to have a higher density, which is also beneficial for achieving oxygen balance [5]. Triazole is the representative of high nitrogen-containing compounds and increasingly attracts considerable interest. In energetic skeletons such as those of imidazole [6], triazole [7], tetrazole [8], triazine [9], and tetrazine [10], 1,2,4-triazole shows unique advantages.

In order to achieve a balance between detonation performance and safety in energetic materials, researchers have employed various methods, including the synthesis of dense ring compounds, the development of eutectic materials, and the preparation of energetic ionic salts. In recent years, energetic salts have drawn widespread attention as a new type of energetic material [11]. Compared to similar molecular compounds, these synthetic products possess more advantages in terms of their basic properties. Energetic salts composed of anions and cations exhibit the characteristics of ionic compounds. These include a low vapor pressure, excellent thermal stability, a high density, and minimal environmental impacts, therefore representing extensive application potential [12]. The studies available in the literature have revealed that the incorporation of methyl groups into the molecular structure of these salts can prove effective in reducing sensitivity and enhancing safety, as well as elevating the decomposition temperature of energetic materials [13,14].

As a nitrogen-rich compound, 4-amino-3-hydrazino-5-methyl-1,2,4-triazole, which brings methyl and 1,2,4-triazole into a single material, has multiple modification sites. This compound can be chemically modified with different groups, leading to enhanced physical and chemical properties when incorporated into ionic salts. Furthermore, the triazole framework allows for the selection of various anions, including those containing nitro groups, which can improve oxygen balance. In contrast to the precursors reported in the literature, which usually have one monovalent anion, the 4-amino-3-hydrazino-5-methyl-1,2,4-triazole described in this article serves as a precursor for polyvalent ion salts, and can be paired with multiple-ion ligands. Polyvalent ions have higher formation heat compared to those of monovalent ion salts with a similar structure. Therefore, the polyvalent state of this invention is related to significant detonation. Taking into consideration the aforementioned advantages, energetic salts with good explosive performances based on 3-hydrazino-4-amino-5-methyl-1,2,4-triazole are reported and comprehensively characterized.

## 2. Results and Discussion

### 2.1. X-ray Crystallography

**Figure 1:**
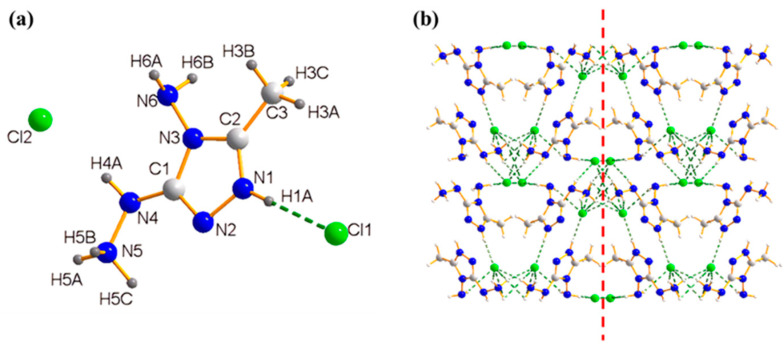
(**a**) The molecular structure and intramolecular hydrogen bonds of **3**; (**b**) The packing diagram and intermolecular weak interactions of **3** (green lines represent hydrogen bonds).

The crystal structure of compound **3** reveals that it belongs to the space group Pbca, with a crystal density of 1.435 g cm^−3^ at 296 K. It can be seen from Figure 1a that one unit of compound **3** consists of two chloridions and one hetercocylic cation, with two protonating hydrogen atoms on N1 and N5. There is one intramolecular interaction between the cation and chloridion (N1-H1A…Cl1), with a distance of 3.066(2) Å and an angle of 177(3)°. Notably, it can surprisingly be found that all of the atoms except the H of the cation are in nearly the same plane, which can be verified by the torsion angles N6-N3-C1-N4 = 5.866°, C3-C2-N3-C1 = 178.976°, and N3-C1-N2-N1 = −0.149°. However, there is a slight deviation in the torsion angle of the hydrazine moiety, with the angle of N5-N4-C1-N3 being −165.121°. In terms of bond lengths, the distance of N3-NH_2_ (1.4013 Å) is shorter than that of N4-NH_2_ (1.4145 Å), and the distance of C2-CH_3_ is the longest among those of all of the bonds in compound **3**, at 1.4639 Å. Additionally, the packing diagram of compound **3** is shown in Figure 1b, from which it can be found that the stacked structures are symmetrical and the different molecules are connected by multiple hydrogen bonds (N4-H4A…Cl2, N5-H5A…Cl2, N5-H5B…Cl1, N5-H5C…Cl1, N6-H6A…Cl1, N6-H6B…Cl2, and N6-H6C…Cl2) to form a stable network structure [15].

**Figure 2:**
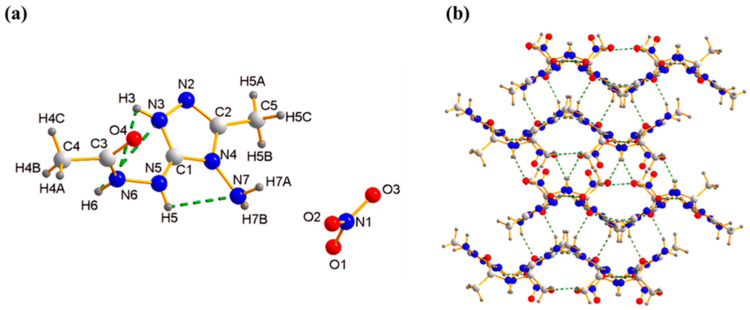
(**a**) The molecular structure and intramolecular hydrogen bonds of **5**; (**b**) The packing diagram and intermolecular weak interactions of **5** (green lines represent hydrogen bonds).

The crystal structure of 4-amino-3-acethydrazino-5-melthy-1,2,4-triazole nitrate energetic compound **5** is depicted in Figure 2. Briefly, **5** belongs to the space group P2 (1)/c with a crystal density of 1.513 g cm^−3^ at 296 K. Each lattice cell contains four molecules (Z = 4) and the molar ratio of the compound **5** is 1:1. The protonating hydrogen atom is on N3. There are three intramolecular interactions in the cation with a distance from 2.5858 Å to 3.1800 Å which can be seen in Figure 2a. The planarity of **5** is close to that of compound **3**, as is evident from the corresponding torsion angles: N5-C1-N3-N2 = 176.068°, N7-N4-C2-N2 = −176.188°, and N4-C2-N2-N3 = −0.109°. However, some torsion angles deviate from the plane, such as O4-C3-N6-N5 = 7.352°, N6-N5-C1-N3 = 15.253° and C3-N6-N5-C1 = −96.923°. For the electron absorption effect of nitrate, we can find that the distance of N4-NH_2_ (1.3993 Å) and the distance of N5-N6 (1.3892 Å) are shorter than those of the compound **3**. Additionally, the distance of C3-CH_3_ (1.4855 Å) is longer than that of C2-CH_3_ (1.4772 Å). Figure 2b displays the combined action of several hydrogen bonds (N3-H3…O1, N5-H5…O1, N6-H6…O2, N7-H7A…O4, N7-H7B…O4, and C4-H4A…O2). All hydrogen bonds consist of cations and oxygen atoms in nitrate ions, and they combine the different molecules tightly, adopting a firm wavelike stacking arrangement.

**Figure 3:**
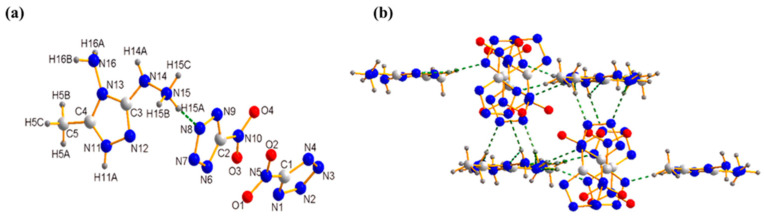
(**a**) The molecular structure and intramolecular hydrogen bonds of **9**; (**b**) The packing diagram and intermolecular weak interactions of **9** (green lines represent hydrogen bonds).

Crystal **9** is presented as (AHMT)^+^ (NT)^−^, with a molar ratio of 1:2. Compound **9** belongs to the triclinic crystal system with the P-1 space group and a crystal density of 1.621g·cm^−3^ at 296K. Each lattice cell contains two molecules (Z = 2). The protonating hydrogen atoms are locked on N11 and N15. One intramolecular interaction (N8-H15A) is shown in Figure 3a with a distance of 1.9045 Å which is shorter than those observed in crystal **3** and **5**. Similarly, the planarity of the cation of compound **9** is similar to those of compounds **3** and **5,** indicating a coplanar structure. This can be confirmed by the torsion angles of C5-C4-N11-N12 = −179.696°, N16-N13-C3-N12 = 177.788°, N15-N14-C3-N12 = −13.543°, and C4-N11-N12-C3 = 0.620°. However, the anion NT is not in the same plane as the cation, with an angle between them of N14-N15-N8 = 100.263°. The distance of N13-NH_2_ (1.3993 Å) is shorter than that of N14-NH_2_ (1.4160 Å), and the distance of C4-CH_3_ is 1.4754 Å. It is can be seen from Figure 3b that there are many intermolecular hydrogen bonds (N11-H11A…N4, N14-H14A…N6, N15-H15B…N7, N15-H15C…N1, N16-H16A…N9, and N16-H16B…N3, C5-H5C…N2) in the packing diagram, and the molecule adopts a face-to-face stacking arrangement.

### 2.2. Physicochemical and Energetic Properties

The thermal stabilities of the above energetic salts were determined using DSC measurements with a scanning rate of 5 °C min^−1^. As shown in Table 1, most of new materials decompose at temperatures below 200 °C varying in the range between 126.8 and 213.7 °C. Among these energetic salts, compound **3** exhibits the highest thermal stability, with a decomposition temperature of 213.7 °C. Compounds **5** and **9** also show high thermal decomposition temperatures of 145.7 °C and 173.4 °C, respectively. The observed high thermal stability can be attributed to the extensive hydrogen bonding interactions between the amino groups of the cation and the oxygen atoms of the anion within these compounds. These strong intermolecular interactions contribute to the overall stability of the materials.

Density is one of the most important evaluation parameters used to measure the performance of energetic materials, because it has a huge effect on their detonation performances [16]. The densities of these salts were measured using Automatic Density Analyzer. The densities of the synthesized salts **3**–**11** are in the range of 1.435–1.989 g cm^−3^, that most of them are higher than that of TNT (1.65 g cm^−3^).

Oxygen balance is a measure of the degree of oxidation in an explosive. Currently, most common explosives have a negative oxygen balance, which means they do not have enough oxidizing agents to completely oxidize the combustible elements. As a result, their detonation performances do not reach their maximum potential [17]. The CO oxygen balance of the energetic salts that were synthesized are listed in Table 1. The oxygen balance of salts **6** (−12.6%), **7** (0%), **8** (0%) and **10** (−16.4%) are much better than that of TNT (−24.7%) and RDX (−21.6%). Compound **7** and **8** are the best in these salts, where the oxygen balance approaches zero, which means that the combustible element of the energetic materials **7** and **8** can be completely oxidized and release maximum heat.

The impact sensitivities of the synthesized energetic salts were measured by using a BAM drop hammer [18]. Compound **3** exhibited excellent sensitivity parameters, with an impact sensitivity (IS) exceeding 40 J, meaning it can be classified as an insensitive energetic material. On the other hand, 5-nitrotetrazolate-2N-oxide salt **10** demonstrated high sensitivity to impact, with a recorded value of 6.1 J. In comparison to RDX, it was found that all the salts, except for 5-nitrotetrazolate salt **9** and 5-nitrotetrazolate-2N-oxide salt **10**, displayed lower sensitivities. This suggests that the majority of the synthesized salts possess reduced sensitivity to impact when compared to that of RDX, which is a desirable characteristic in terms of safety and stability. 

EXPLO5 V6.01 was used to calculate the detonation velocities and pressures with the data of heats of formation, formula and density [19,20]. The detonation velocities fall in the range of 6600–8887 m s^−1^ and the detonation pressures are in the range of 17.0–34.2 GPa. These values indicate significantly improved performance compared to that of TNT (6881 m s^−1^, 19.5 GPa) with the exception of compound **3**. The oxygen-balanced explosives **7** and **8** show the best performance; **7** has the highest detonation pressure (34.2 GPa) and **8** has the highest detonation velocity (8887 m s^−1^), which is comparable to that of RDX.

### 2.3. 2D Fingerprints and Hirshfeld Surface Analysis

**Figure 4:**
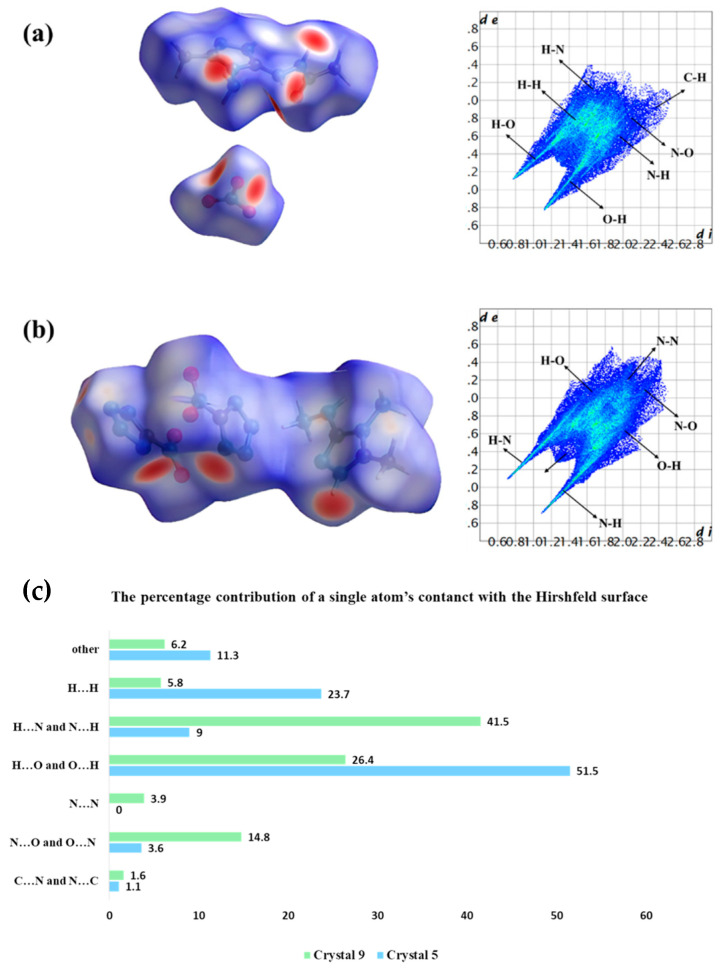
(**a**) The Hirschfeld surface and 2D fingerprint plot of **5**; (**b**) The Hirschfeld surface and 2D fingerprint plot of **9**; (**c**) The percentage contribution of a single atom’s contact with the Hirshfeld surfaces.

To investigate the relationship between the molecular structure of the compounds and their sensitivity, Hirschfeld surfaces and 2D fingerprint plots are used to analyze the intra- and intermolecular interactions of compounds **5** and **9** [21,22]. There are some red dots observed in the Hirshfeld surfaces that can be seen from Figure 4a,b indicating the presence of intermolecular hydrogen bonding. These hydrogen bonding interactions include H…O and O…H, and N…H and H…N interactions. Additionally, it is easy to observe from the 2D fingerprint plots that there are two spikes on the left of the picture which represent the hydrogen bonds of compounds **5** and **9**, but the types of the hydrogen bonds of the spikes of the two plots are different, being H…O and O…H interactions in Figure 4a and N…H and H…N interactions in Figure 4b. The bule aspects represent the weaker connection of the compound compared to that in the red regions. The 2D fingerprint plots provide information about the distributions of the different intermolecular interactions in these compounds which are shown in Figure 4c. Compounds **5** and **9** have 60.5% and 68.1% hydrogen bonds, respectively. Additionally, we can find that the percentage of π–π interactions (C…N and N…C; N…O, O…N and N…N) of compounds **5** and **9** are 4.7% and 20.3%, respectively. We can find that hydrogen bonds play the dominant role. Because hydrogen bonds stabilize the compound under external stimuli, it is easy to understand why compounds **5** and **9** are stable.

### 2.4. Noncovalent Interaction (NCI) Analysis

**Figure 5:**
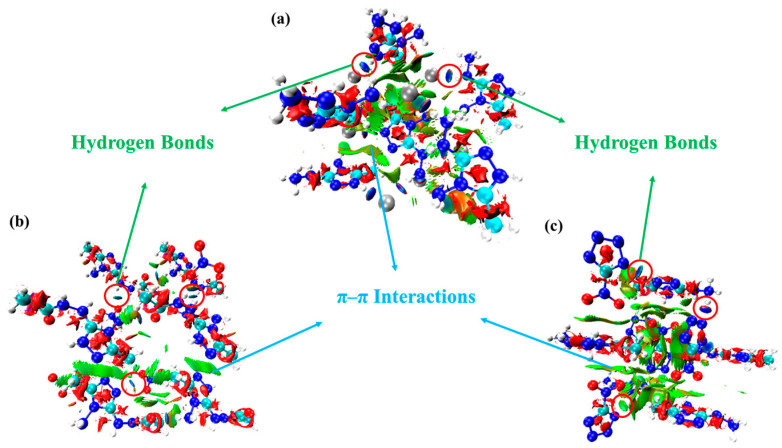
Noncovalent interaction analysis for **3** (**a**), **5** (**b**) and **9** (**c**).

The noncovalent interactions result of the compounds **3**, **5** and **9** are presented in Figure 5 [23,24,25]. In the figure, we can see that there are three different colors; blue represents a strong interaction, green represents a weak interaction and red represents strong repulsion. It is easy to see the blue round ellipsoids presented in Figure 5a–c which mean that **3**, **5** and **9** have the hydrogen bonds, and which is consistent with the results of the 2D fingerprints and Hirshfeld surface analysis. Additionally, we find that there are many green planes in the figure, that means the compounds have π–π interactions. Notably, we find that there are more green planes representing π–π accumulations in Figure 5c than in Figure 5b, which corresponds with the results of the percentage contribution of the single atom making contact with the Hirshfeld surfaces in Figure 4c. In addition, we find a few red ellipsoids near 1,2,4-triazole and C-CH_3_.

### 2.5. Electrostatic Potential Surface (ESP) Analysis

**Figure 6:**
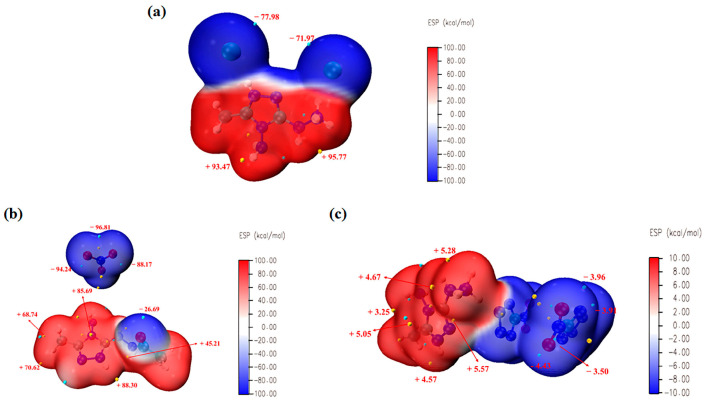
Electrostatic potential of compounds **3** (**a**), **5** (**b**) and **9** (**c**).

To gain further insights into the relationship between different substituents and molecular sensitivity, the electrostatic potential surface analysis (ESP) of compounds **3**, **5** and **9** were conducted [24,25,26]. In ESP analysis, the minimum and maximum values are represented by blue and red points, respectively. The red and blue areas indicate electropositive and electronegative regions. As shown in the following Figure 6a, the minimum value (−77.98 kcal/mol) of **3** arises from the atom of Cl and the maximum value (95.77 kcal/mol) of **3** arises from hydrazine. Moving on to compound **5**, we can find that the minimum value (−96.81 kcal/mol) is attributed to the nitrate group and the maximum value of it arises from triazole which is 88.30 kcal/mol. We find that the electrostatic value of C=O in the cation is negative at −26.69 kcal/mol, as shown in Figure 6b. In contrast, the values for compound **9** are much smaller than those of compounds **3** and **5**, with a minimum value of −4.43 kcal/mol and a maximum value of 5.57 kcal/mol. As commonly known, the lower the maximum ESP values and the greater the negative charge accumulation in the nitro-substituent, the more stable the compound.

## 3. Materials and Methods

**Scheme 1:**
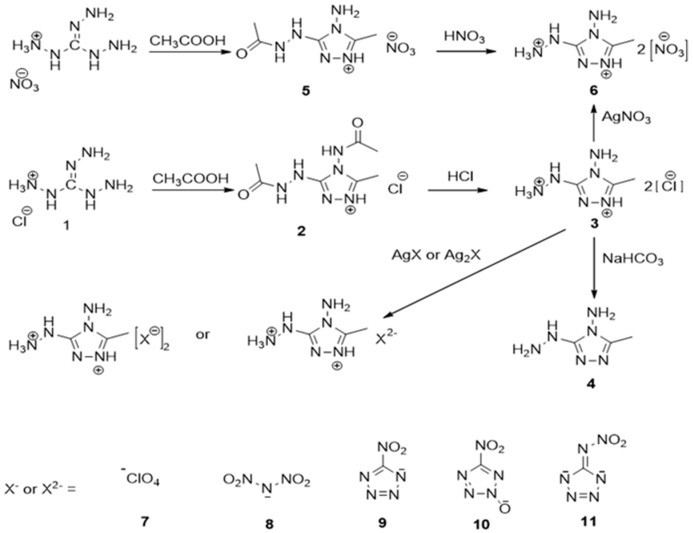
Synthesis of compound **1**–**11**.

The synthesis route for 4-amino-3-hydrazino-5-methyl-1,2,4-triazole and its salts, those described in this study, is illustrated in Scheme 1. Guanidine hydrochloride was added into 3 equivalents of hydrazine hydrate, refluxing at 110 °C for 2 h, yielding triaminoguanidine hydrochloride **1** (TAG·HCl). After reacting with acetic acid, the solvent was removed and produced a viscous, resinous residue. The reaction mixture was then treated with acetic acid, and the solvent was evaporated to yield a viscous, resinous residue. From this residue, a moderate yield of 4-acetamido-3-acethydrazino-5-methyl-1,2,4-triazole hydrochloride **2** was isolated.

As depicted in Scheme 1, the combination of the nitrogen-rich divalent cation (AHMT^2+^) with nitrogen-rich anions forms a class of energetic materials, whose energy is derived from their positive heats of formation as well as the combustion of the carbon atoms. Compounds **6**–**11** were readily synthesized via anion exchange with silver nitrate, silver perchlorate, silver dinitramide, silver 5-nitrotetrazolate, silver 5-nitrotetrazolate-2N-oxide, and silver 5-nitroiminotetrazolate, respectively, with **3** in methanol or water. 4-amino-3-hydrazino-5-methyl-1,2,4-triazolium **4** was isolated by treating **3** with stoichiometric amounts of NaHCO_3_. Additionally, salt **6** could be synthesized by refluxing TAG·HNO_3_ in acetic acid, followed by hydrolysis with dilute nitric acid. 

### 3.1. Experimental Section

The instruments and setting parameters used in the preparation and analysis are available in the general methods included in the Supporting Information.

#### 3.1.1. Caution

Although we did not meet any danger in the process of these experiments, some necessary safeguard procedures were carried out such as the use of a hood, a pair of goggle and leather gloves. The risk of making contact with any of these materials directly should be avoided. Due to the positive heats of formation, extreme caution should be exercised in the performance of synthesis and characterization.

#### 3.1.2. Synthesis

For 4-Amino-3-hydrazino-5-methyl-1,2,4-triazolium hydrochloride (**3**), Triaminoguanidine hydrochloride (TAG·HCl) (2.81 g, 20 mmol) was added to 15 mL of acetic acid. The mixture was stirred and refluxed at 100–110 °C for 2 h. The excess volatile acetic acid was removed under a partial vacuum to recover a viscous, resinous residue. From this residue, 4-acetamido-3-acethydrazino-5-methyl-1,2,4-triazole hydrochloride (**2**) was isolated in a moderate yield. The viscous residue was dissolved and stirred vigorously in diluted hydrochloric acid at 80 °C. As the reaction progressed, white crystals of 4-Amino-3-hydrazino-5-methyl-1,2,4-triazolium hydrochloride (**3**) precipitated. The product was filtered and washed with several aliquots (50 mL total) of ice water. The product was dried under high vacuum, resulting in a good yield (2.13 g, 53%) of **3**. MS *m*/*z* (ESI^+^): 129.08 [C_3_H_9_N_6_^+^]; elemental analysis (%) Calc. for C_3_H_10_N_6_Cl_2_ (MW = 201.06 g·mol^−1^): C, 17.91; H, 4.98; N, 41.79; found C, 17.85; H, 5.05; N, 41.27; IR (KBr): 3182, 2836, 2659, 1943, 1704, 1609, 1569, 1541, 1484, 1423, 1377, 1322, 1263, 1228, 1165, 1141, 1092, 1046, 995, 966, 822, 750, 711, 616, 553 cm^−1^; ^1^H NMR (DMSO) δ: 9.92, 9.31; ^13^C NMR (DMSO) δ: 156.95, 153.45 ppm.

4-Amino-3-hydrazino-5-methyl-1,2,4-triazolium (**4**): Compound 3 (2.01 g, 10 mmol) was dissolved in 30 mL of distilled water. While stirring, 16.8 mL of sodium bicarbonate (10 wt%) was added dropwise. The colorless solutions turned purple. The mixture was stirred for 30 min and evacuated under vacuum to produce a purple mixture. The mixture was dissolved in 25 mL of absolute methanol and the inorganic salt was removed via filtration. After filtering, the solvent was removed under reduced pressure to produce a purple solid of **4** at a 73% (0.934 g) yield. MS *m*/*z* (ESI^+^): 129.1 [C_3_H_9_N_6_^+^]; elemental analysis (%) calcd. for C_3_H_8_N_6_ (MW = 128.14 g·mol^−1^): C 28.13, H 6.25, N 65.63; found C 28.07, H 6.37, N 64.73; IR (KBr): 3344, 3215, 3026, 2843, 2661, 1965, 1620, 1585, 1526, 1486, 1254, 1232, 1091, 1051, 997, 835, 742, 654 cm^−1^; ^1^H NMR (DMSO) δ: 9.62, 9.15, 8.26 ppm; ^13^C NMR (DMSO) δ: 174.35, 152.25, 20.0 ppm.

General procedure for the preparation of energetic salts **6**–**11**.

A solution of silver nitrate, silver perchlorate, silver dinitramide, silver 5-nitrotetrazolate, and silver 5-nitrotetrazolate-2N-oxide (10 mmol) in distilled water (20 mL), or silver 5-nitroiminotetrazolate (5 mmol) in distilled water (10 mL) was added dropwise to the solution of compound 11 (1.005 g, 5 mmol) in distilled water (20 mL). After stirring at room temperature for 1 h, the precipitate was filtered and rinsed with 10 mL of distilled water. The solvent was evaporated in vacuum and the residue was recrystallized from methanol and dried in air to obtain the target product.

4-Amino-3-hydrazino-5-methyl-1,2,4-triazolium nitrate (**6**): Yield: 96%, white crystals. MS *m*/*z* (ESI^+^): 129.1 [C_3_H_9_N_6_^+^]; elemental analysis (%) calcd for C_3_H_10_N_8_O_6_ (MW = 254.16 g·mol^−1^): C 14.17, H 3.94, N 44.09; found C 14.21, H 4.15, N 43.38; IR (KBr): 3319, 2847, 2724,1643, 1614, 1558, 1418, 1293, 1234,1159, 1077, 1051, 1036, 966, 947, 867, 815, 720, 702, 637, 618, 566 cm^−1^; ^1^H NMR(DMSO) δ: 10.30, 9.77, 6.08 ppm; ^13^C NMR (DMSO) δ: 169.99, 152.89 ppm.

4-Amino-3-hydrazino-5-methyl-1,2,4-triazolium perchlorate (**7**): Yield: 90%, colorless transparent crystals. MS *m*/*z* (ESI^+^): 129.1 [C_3_H_9_N_6_^+^]; elemental analysis (%) calcd for C_3_H_10_N_6_Cl_2_O_8_ (MW = 329.05 g·mol^−1^): C 10.94, H 3.04, N 25.53; found C 10.89, H 3.12, N 26.27; IR (KBr): 3327, 3131, 2989, 2695, 1645, 1536, 1455, 1421, 1320, 1235, 1185, 1039, 946, 835, 712, 668, 555 cm^−1^; ^1^H NMR(DMSO) δ: 10.41, 9.55 8.43 ppm; ^13^C NMR (DMSO) δ: 152.53, 142.34 ppm.

4-Amino-3-hydrazino-5-methyl-1,2,4-triazolium dinitramide (**8**): Yield: 88%, white solid. Elemental analysis (%) calcd for C_3_H_10_N_12_O_8_ (MW = 342.19 g·mol^−1^): C 10.53, H 2.92, N 49.12; found C 10.61, H 3.09, N 48.82; IR (KBr): 3326, 3162, 3148, 2412, 1932, 1774, 1565, 1412, 1393, 1318, 1285, 1261, 1219, 1086, 1048, 1005, 929, 885, 848, 757, 731, 671, 639, 557 cm^−1^; ^1^H NMR(MeOD) δ: 10.82, 9.87, 6.79 ppm; ^13^C NMR (CD_4_O) δ: 153.33, 144.34, 143.33 ppm.

4-Amino-3-hydrazino-5-methyl-1,2,4-triazolium 5-nitrotetra-zolate (**9**): Yield: 93%, pale yellow solid. MS *m*/*z* (ESI^+^): 129.1 [C_3_H_9_N_6_^+^]; *m*/*z* (ESI^−^): 114.0 [CN_5_O_2_^−^]; elemental analysis (%) calcd for C_5_H_10_N_16_O_4_ (MW = 358.24 g·mol^−1^): C 16.76, H 2.79, N 62.57; found C 17.01, H 3.82, N 61.30; IR(KBr): 3355, 2935, 2692, 2100, 1736, 1667, 1608, 1504, 1450, 1320, 1269, 1228, 1187, 1170, 1148, 1106, 1069, 1056, 1039, 772, 746, 710, 615, 557 cm^−1^; ^1^H NMR (DMSO) δ: 9.91, 9.38 ppm; ^13^C NMR (DMSO) δ: 169.33, 157,28, 153.63, 151.31 ppm.

4-Amino-3-hydrazino-5-methyl-1,2,4-triazolium 5-nitrotetra-zolate-2N-oxide (**10**): Yield: 86%, pale yellow solid. MS *m*/*z* (ESI^+^): 129.1 [C_3_H_9_N_6_^+^]; elemental analysis (%) calcd for C_5_H_10_N_16_O_6_ (MW = 390.24 g·mol^−1^): C 15.38, H 2.56, N 57.44; found C 15.89, H 2.61, N 56.91; IR(KBr) ν: 3347, 3256, 3121, 3071, 2932, 2789, 1855, 1692, 1585, 1541, 1437, 1406, 1309, 1249, 1197, 1141, 1082, 1057, 996, 965, 934, 837, 775, 709, 638, 553 cm^−1^; ^1^H NMR(DMSO) δ: 10.83, 9.97, 8.28 ppm; ^13^C NMR(DMSO) δ: 161.63, 156.25, 155.13 ppm.

4-Amino-3-hydrazino-5-methyl-1,2,4-triazolium 5-nitroimino-tetrazolate (**11**): Yield: 83%, white solid. MS *m*/*z* (ESI^+^): 129.0 [C_3_H_9_N_6_^+^]; *m*/*z* (ESI^−^): 127.98 [CN_6_O_2_^−^]; elemental analysis (%) calcd for C_4_H_10_N_12_O_2_ (MW = 258.2 g·mol^−1^): C 18.60, H 3.88, N 65.12; found C 18.48, H 3.81, N 64.28; M.p. 164.5 °C; IR(KBr): 3440, 3348, 3277, 3090, 2978, 2901, 2733, 2679, 1709, 1651, 1599, 1540, 1441, 1328, 1268, 1234, 1158, 1063, 1033, 999, 869, 836, 685, 504, 425 cm^−1^; ^1^H NMR(DMSO) δ: 10.33, 9.13, 7.83, 7.68 ppm; ^13^C NMR(DMSO) δ: 157.32, 156.35, 150.37, 25.37 ppm.

## 4. Conclusions

In this study, we successfully synthesized a new precursor, 4-amino-3-hydrazino-5-methyl-1,2,4- triazolium hydrochloride **3** (AHMT·2HCl). The nitrogen-rich compound **3** was synthesized via the reaction of triaminoguanidine hydrochloride and acetic acid and then hydrolyzed with diluted hydrochloric acid. Via Brønsted acid–base reactions, a series of energetic salts based on compound **3** were synthesized and fully characterized. The structures of **3**, **5** and **9** were confirmed via X-ray single-crystal diffraction. Cations can be either polyvalent, monovalent or bivalent. Among all energetic salts based on 4-amino-3-hydrazino-5-methyl-1,2,4-triazole, compound **3** shows the best stability, and dinitramide salt **8** shows excellent properties with a detonation pressure of 33.9 GPa, a detonation velocity of 8887 m s^−1^ and an optimal oxygen-balance state. Therefore, considering its excellent performance and desirable properties, compound **8** holds great potential for utilization in various areas within the energetic materials field.

## Figures and Tables

**Table 1:** The physicochemical properties of related energetic salts compared to those of TNT and RDX.

Salt	T_d_ ^a^	T_sd_ ^b^	d_c_ ^c^/d_m_ ^d^	OB ^e^	$\Delta H_{f}^{o}$ ${\;}^{f}/\Delta H_{m}^{o}{\;}^{g}$	P ^h^	D ^i^	IS ^j^
**3**	213.7	-	1.445/1.435	−64	246.79/1.23	17.0	6600	>40
**5**	145.7	232.9	1.521/1.513	−44.61	374.59/1.61	25.1	7705	31.6
**6**	163.8	302.6	1.69/1.679	−12.6	−280.5/−1.10	25.6	8170	16.3
**7**	126.8	266.5	2.01/1.989	0.0	−291.1/−0.88	34.2	8528	28.9
**8**	164.6	231.1	1.78/1.791	0.0	177.4/0.52	33.9	8887	10.7
**9**	173.4	233.2	1.69/1.621	−26.8	697.8/1.95	23.6	8031	7.1
**10**	183.2	234.8	1.73/1.712	−16.4	676.4/1.73	28.6	8524	6.1
**11**	173.9	-	1.65/1.652	−43.4	354.4/1.37	22.6	8039	>40
TNT	295.0	-	1.65	−24.7	−67/−0.295	19.5	6881	15
RDX	230	-	1.82	−21.6	83.8/0.38	35.2	8977	7.4

^a^ Decomposition temperature (°C). ^b^ Secondary decomposition temperature (°C). ^c^ Calculated density (g·cm^−3^). ^d^ Measured density (g·cm^−3^). Use of ULTRAPYC 1200. ^e^ CO_2_ oxygen balance (OB) is an index of the deficiency or excess of oxygen in a compound required to convert all C atoms into CO and all H atoms into H_2_O. For a compound with the molecular formula of C_a_H_b_N_c_O_d_ (without crystal water), OB (%) = 1600[(d-a-b/2)/MW], where MW is the molecular weight of salt. ^f^ Molar enthalpy of the formation of salt (kJ·mol^−1^). ^g^ Enthalpy of the formation of ionic salts in per gram (kJ·g^−1^). ^h^ Detonation pressure (GPa). ^i^ Detonation (m·s^−1^). ^j^ Impact sensitivity (J).

## Data Availability

Not applicable.

## Supplementary Materials

The following supporting information can be downloaded at. https://www.mdpi.com/article/10.3390/ijms241713136/s1. References [19,20,21,22,23,24,25,26] are cited in there.
