# Supplementary material for: Polyvalent Ionic Energetic Salts Based on 4-Amino-3-hydrazino-5-methyl-1,2,4-triazole
Source: Int J Mol Sci. 2023 Aug 24;24(17):13136. doi: 10.3390/ijms241713136 (PMC10487886; doi:10.3390/ijms241713136)
Supplement: Supplementary file 1 [file ijms-24-13136-s001.zip › ijms-2547959-supplementary.pdf]

Supporting Information

Polyvalent ionic energetic salts based on 4-amino- 3-hydrazino-5-methyl-1,2,4-triazole

Qiuhan Lin [*†], Mingmin Zhang[†], Linan Zhang, Mimi Zhu, Kaiyi Qin, Pengcheng Wang [*]

School of Chemistry and Chemical Engineering, Nanjing University of Science and Technology, Nanjing 210094, China; zmm1219@njust.edu.cn (M.Z.); zhanglinan2019@njust.edu.cn (L.Z.); 121103022194@njust.edu.cn (M.Z.); qinky0731@njust.edu.cn (K.Q.)

* linqh@njust.edu.cn (Q.L.); alxwpch@njust.edu.cn (P.W.)

† These authors contributed equally to this work.

Table of Contents

1. Crystal data and structure refinement for 3, 5 and 9

2. Optimized structures and Cartesian coordinates of 3-hydrazino-4-amino-5-melthyl-1,2,4-triazolium (AHMT) cation

3. Heat of formation and densities calculations of AHMT based salts

4. Method of calculated densities

5. Sensitivity

6. General methods

7. X-ray Crystallographic Studies

8. Theoretical study

9. ^{1}H Spectra of related compounds

10. ^{13}C Spectra of related compounds

11. IR Spectra of related compounds

12. DSC thermogram of the related compounds

13. References

1. Crystal data and structure refinement for **3, 5** and **9**

Table S1. Crystal data and structure refinement for **3, 5** and **9**.

Crystal	**3**	**5**	**9**
Empirical formula	$C_3H_{10}N_6Cl_2$	$C_5H_{11}N_7O_4$	$C_5H_{10}N_{16}O_4$
Temperature	296(2)K	296K	297(2)K
Wavelength	0.71073 A	0.71073 A	0.71073 A
Crystal system	Orthorhombic	monoclinic	Triclinic
Space group	*Pbca*	P2(1)/c	P-1
Unit cell dimensions	*a* = 12.2316(13)Å, *α*= 90.00°<br>*b* = 7.6047(9) Å, *β*= 90.00(6)°<br>*c* = 20.010 Å, *γ*= 90.00°	a=10.1097(11) Å, α= 90.00°<br>b=7.8327(8) Å, β= 105.008(4)°<br>c=13.3872(14) Å, γ= 90.00°	a = 7.8831(6) Å, α=88.597(2) °<br>b = 9.5579(6) Å, β= 77.318(2) °<br>c = 10.5053(7) Å, γ= 72.096(2) °
Volume	1861.3(3) $Å^3$	1023.92(19)	734.04(9) Å3
Z	8	4	2
Calculated density	1.435 $g{\cdot}cm^{-3}$	1.513 $g{\cdot}cm^{-3}$	1.621$g{\cdot}cm^{-3}$
Absorption coefficient	0.651 mm^{-1}	0.130 mm^{-1}	0.138 mm^{-1}
F (000)	832	488	368
Crystal size	0.23 x 0.20 x 0.08 mm	0.22 x 0.20 x 0.11 mm	0.25 x 0.22 x 0.14 mm
Theta range for data collection	3.31° to 25.35°	3.15° to 25.39°	2.99° to 25.41°
Limiting indices	-14≤*h*≤13, -9≤*k*≤9, -24≤*l*≤21	-12≤*h*≤11, 0≤*k*≤9, 0≤*l*≤15	-9≤h≤9, -11≤k≤11, -12≤l≤12
Reflections collected / unique	10081/1700 [*R*(*int*) = 0.0683]	10081/1700 [*R*(*int*) = 0.0744]	6664/2676 [R(int) = 0.0244]
Completeness to theta = 27.73	99.5%	99.5%	98.3%
Max. and min. transmission	0.7452 and 0.6663	0.745 and 0.615	0.7452 and 0.6359
Reflections with I>2σ (I)	1206	1257	1720
Goodness-of-fit on F2	1.056	1.050	1.010
Final R indices (I>2sigma(I))	*R1* = 0.0363, *wR2* = 0.0751	*R1* = 0.0744, *wR2* = 0.1376	R1 = 0.0448, wR2 = 0.1097
R indices (all data)	*R1* =0.0688, *wR2* = 0.0845	*R1* =0.1431 *wR2* = 0.1679	R1 = 0.0876, wR2 = 0.1285
CCDC	1516715	2281718	1516716

Table S2. Bond lengths [Å] and angles [°] for **3**.

N(1)-N(2)	1.383(3)
N(1)-C(2)	1.306(4)
N(2)-C(1)	1.299(3)
N(3)-N(6)	1.402(3)
N(3)-C(1)	1.357(3)
N(3)-C(2)	1.343(3)
N(4)-N(5)	1.414(3)
N(4)-C(1)	1.366(3)
N(1)-H(1A)	0.88(3)
C(2)-C(3)	1.464(4)
N(4)-H(4A)	0.85(2)
N(5)-H(5A)	0.94(2)
N(5)-H(5B)	0.94(2)
N(5)-H(5C)	0.99(2)
N(6)-H(6A)	0.88(2)
N(6)-H(6B)	0.86(2)
C(3)-H(3A)	0.9600
C(3)-H(3B)	0.9600
C(3)-H(3C)	0.9600
N(2)-N(1)-C(2)	113.7(2)
N(1)-N(2)-C(1)	101.7(2)
N(6)-N(3)-C(1)	123.6(2)
N(6)-N(3)-C(2)	129.2(2)
C(1)-N(3)-C(2)	107.2(2)
N(5)-N(4)-C(1)	114.93(19)
N(2)-C(1)-N(3)	112.5(2)
N(2)-C(1)-N(4)	126.2(2)
N(3)-C(1)-N(4)	121.2(2)
N(2)-N(1)-H(1A)	120.3(19)
C(2)-N(1)-H(1A)	126.1(19)
N(3)-C(2)-C(3)	126.9(2)
N(1)-C(2)-N(3)	105.0(2)
N(1)-C(2)-C(3)	128.2(2)
N(5)-N(4)-H(4A)	112.1(16)
C(1)-N(4)-H(4A)	115.4(15)
N(4)-N(5)-H(5A)	109.7(14)

N(4)-N(5)-H(5B)	109.2(14)
N(4)-N(5)-H(5C)	113.1(14)
H(5A)-N(5)-H(5B)	111.5(19)
H(5A)-N(5)-H(5C)	106.6(19)
H(5B)-N(5)-H(5C)	106.8(18)
N(3)-N(6)-H(6A)	106.9(17)
N(3)-N(6)-H(6B)	106.1(19)
H(6A)-N(6)-H(6B)	110(3)
C(2)-C(3)-H(3A)	110.00
C(2)-C(3)-H(3B)	109.00
C(2)-C(3)-H(3C)	109.00
H(3A0-C(3)-H(3B)	109.00
H(3A)-C(3)-H(3C)	109.00
H(3B)-C(3)-H(3C)	109.00

Symmetry transformations used to generate equivalent atoms:

Table S3. Hydrogen bonds for **3** [Å and °].

D-H...A	d(D-H)	d(H...A)	d(D...A)	<(DHA)
N(1)-H(1A)...Cl(1)	0.88(3)	2.19(3)	3.066(2)	177(3)
N(4)-H(4A)...Cl(2)#	0.85(2)	2.26(2)	3.084(2)	161(2)
N(5)-H(5A)...Cl(2)#	0.94(2)	2.16(2)	3.083(2)	167.1(19)
N(5)-H(5B)...Cl(1)#	0.94(2)	2.21(2)	3.120(2)	163.3(18)
N(5)-H(5C)...Cl(1)#	0.99(2)	2.19(2)	3.128(2)	156.5(19)
N(6)-H(6A)...Cl(1)#	0.88(2)	2.66(3)	3.355(2)	136(2)
N(6)-H(6B)...Cl(2)#	0.86(2)	2.51(2)	3.204(2)	139(3)
N(6)-H(6B)...Cl(2)#	0.86(2)	2.77(3)	3.319(2)	124(2)

Symmetry transformations used to generate equivalent atoms:

#1 x,y,z #2 1/2-x,-y,1/2+z #3 1/2+x,1/2-y,-z #4 -x,1/2+y,1/2-z #5 -x,-y,-z
#6 1/2+x,y,1/2-z #7 1/2-x,1/2+y,z #8 x,1/2-y,1/2+z

Table S4. Bond lengths [Å] and angles [°] for **5**.

O(4)-C(3)	1.221(5)
N(2)-N(3)	1.390(6)
N(2)-C(2)	1.299(6)
N(3)-C(1)	1.308(6)
N(4)-N(5)	1.399(5)
N(4)-C(1)	1.344(5)
N(4)-C(2)	1.382(5)
N(5)-N(6)	1.389(5)
N(5)-C(1)	1.331(5)
N(6)-C(3)	1.335(5)
O(1)-N(1)	1.242(6)
O(2)-N(1)	1.231(6)
C(2)-C(5)	1.455(5)
N(3)-H(3)	0.82(5)
C(3)-C(4)	1.486(6)
O(3)-N(1)	1.224(6)
N(5)-H(5)	0.80(5)

N(6)-H(6)	0.89(5)
N(7)-H(7A)	0.86(5)
N(7)-H(7B)	0.86(5)
C(4)-H(4C)	0.9600
C(4)-H(4A)	0.9600
C(4)-H(4B)	0.9600
C(5)-H(5A)	0.9600
C(5)-H(5B)	0.9600
C(5)-H(5C)	0.9600

N(3)-N(2)-C(2)	104.1(4)
N(2)-N(3)-C(1)	111.7(4)
N(7)-N(4)-C(1)	121.5(4)
N(7)-N(4)-C(2)	131.4(3)
C(1)-N(4)-C(2)	105.0(3)
N(6)-N(5)-C(1)	118.3(4)
N(5)-N(6)-C(3)	120.0(4)
N(3)-C(1)-N(4)	106.5(4)
N(3)-C(1)-N(5)	129.4(4)
N(4)-C(1)-N(5)	124.0(4)
N(2)-C(2)-N(4)	110.6(4)
N(2)-C(2)-C(5)	126.8(4)
N(4)-C(2)-C(5)	122.6(4)
O(4)-C(3)-N(6)	122.0(4)
O(4)-C(3)-C(4)	122.6(4)
N(6)-C(3)-C(4)	115.4(4)
N(2)-N(3)-H(3)	116(4)
C(1)-N(3)-H(3)	132(4)
N(6)-N(5)-H(5)	115(4)
C(1)-N(5)-H(5)	120(3)
N(5)-N(6)-H(6)	115(3)
C(3)-N(6)-H(6)	125(3)
N(4)-N(7)-H(7A)	108(3)
N(4)-N(7)-H(7B)	105(3)
H(7A)-N(7)-H(7B)	114(5)
O(2)-N(1)-O(3)	120.7(4)
O(1)-N(1)-O(2)	118.9(4)
O(1-N(1)-O(3)	120.4(4)
C(3)-C(4)-H(4B)	109.00

H(4A)-C(4)-H(4C)	109.00
C(3)-C(4)-H(4C)	109.00
H(4A)-C(4)-H(4B)	110.00
C(3)-C(4)-H(4A)	109.00
H(4B)-C(4)-H(4C)	109.00
C(2)-C(5)-H(5C)	110.00
H(5A)-C(5)-H(5C)	109.00
H(5B)-C(5)-H(5C)	109.00
H(5A)-C(5)-H(5B)	109.00
C(2)-C(5)-H(5A)	109.00
C(2)-C(5)-H(5B)	109.00

Symmetry transformations used to generate equivalent atoms:

Table S5. Hydrogen bonds for **5** [Å and °].

D-H...A	d(D-H)	d(H...A)		
N(3)-H(3)...O(1)#	0.82(5)	2.06(5)	2.762(6)	144(5)
N(5)-H(5)...O(1)#	0.80(5)	2.17(5)	2.845(5)	142(4)
N(6)-H(6)...O(2)#	0.89(5)	2.01(5)	2.871(6)	163(4)
N(5)-H(7A)...O(4)#	0.86(5)	2.08(5)	2.876(5)	155(4)
N(5)-H(7B)...O(4)#	0.86(5)	2.08(5)	2.936(5)	174(4)
C(4)-H(4A)...O(2)#	0.9600	2.4800	3.358(6)	155

Symmetry transformations used to generate equivalent atoms:

#1 x,y,z #2 -x,1/2+y,1/2-z #3 -x,-y,-z #4 x,1/2-y,1/2+z

Table S6. Bond lengths [Å] and angles [°] for **9**.

N(11)-N(12)	1.390(3)
N(11)-C(4)	1.295(3)
N(12)-C(3)	1.310(3)
N(13)-N(16)	1.399(4)
N(13)-C(3)	1.360(3)
N(13)-C(4)	1.352(3)
N(14)-N(15)	1.416(3)
N(14)-C(3)	1.353(3)
O(1)-N(5)	1.215(4)
C(4)-C(5)	1.475(3)
N(11)-H(11A)	0.83(2)
N(14)-H(14A)	0.86(3)
N(15)-H(15A)	0.964(19)
N(15)-H(15C)	0.96(2)
N(15)-H(15B)	0.961(19)
N(16)-H(16B)	0.86(3)
N(16)-H(16A)	0.91(3)
N(1)-N(2)	1.345(3)
N(1)-C(1)	1.312(3)
N(2)-N(3)	1.323(3)

N(3)-N(4)	1.341(3)
N(4)-C(1)	1.321(3)
C(5)-H(5A)	0.9600
C(5)-H(5B)	0.9600
C(5)-H(5C)	0.9600
N(5)-C(1)	1.439(4)
O(3)-N(10)	1.207(4)
O(4)-N(10)	1.217(4)
N(6)-N(7)	1.332(3)
N(6)-C(2)	1.319(3)
N(8)-N(9)	1.336(3)
N(9)-C(2)	1.325(4)
N(10)-C(2)	1.442(4)

N(12)-N(11)-C(4)	114.0(2)
N(11)-N(12)-C(3)	101.53(19)
N(16)-N(13)-C(3)	122.57(19)
N(16)-N(13)-C(4)	130.36(19)
C(3)-N(13)-C(4)	107.04(19)
N(15)-N(14)-C(3)	116.3(2)
N(12)-C(3)-N(13)	112.20(19)
N(12)-C(3)-N(14)	126.9(2)
N(13)-C(3)-N(14)	120.8(2)
N(11)-C(4)-N(13)	105.22(19)
N(11)-C(4)-C(5)	128.0(2)
N(13)-C(4)-C(5)	126.8(2)
N(12)-N(11)-H(11A)	119.8(16)
C(4)-N(11)-H(11A)	125.8(16)
N(15)-N(14)-H(14A)	114.0(16)
C(3)-N(14)-H(14A)	118.2(14)
N(14)-N(15)-H(15A)	107.9(15)
N(14)-N(15)-H(15B)	111.7(13)
N(14)-N(15)-H(15C)	108.6(15)
H(15A)-N(15)-H(15B)	113(2)
H(15A)-N(15)-H(15C)	108.3(19)
H(15B)-N(15)-H(15C)	108(2)
N(13)-N(16)-H(16A)	110(2)
N(13)-N(16)-H(16B)	107(2)
H(16A)-N(16)-H(16B)	112(3)

N(2)-N(1)-C(1)	103.7(2)
N(1)-N(2)-N(3)	109.0(2)
N(2)-N(3)-N(4)	109.7(2)
N(3)-N(4)-C(1)	103.3(2)
H(5A)-C(5)-H(5C)	109.00
H(5B)-C(5)-H(5B)	109.00
C(4)-C(5)-H(5A)	109.00
C(4)-C(5)-H(5B)	109.00
C(4)-C(5)-H(5C)	109.00
O(1)-N(5)-O(2)	125.4(3)
O(1)-N(5)-C(1)	117.0(2)
O(2)-N(5)-C(1)	117.5(2)
N(1)-C(1)-N(4)	114.4(2)
N(1)-C(1)-N(5)	122.5(2)
N(4)-C(1)-N(5)	123.1(2)
N(7)-N(6)-C(2)	103.1(2)
N(6)-N(7)-N(8)	109.69(19)
N(7)-N(8)-N(9)	110.0(2)
N(8)-N(9)-C(2)	102.5(2)
O(3)-N(10)-O(2)	117.9(2)
O(4)-N(10)-C(2)	117.7(2)
N(6)-C(2)-N(9)	114.7(2)
N(6)-C(2)-N(10)	122.3(2)
N(9)-C(2)-N(10)	122.9(2)

Symmetry transformations used to generate equivalent atoms:

Table S7. Hydrogen bonds for **9** [Å and °].

D-H...A	d(D-H)	d(H...A)	d(D...A)	<(DHA)
N(11)-H(11A)...N(4)#	0.83(2)	2.13(2)	2.903(3)	155(2)
N(14)-H(14A)...N(6)#	0.86(3)	2.08(3)	2.897(3)	158(2)
N(15)-H(15A)...N(8)	0.964(19)	1.90(2)	2.846(3)	165(2)
N(15)-H(15B)...N(7)#	0.961(19)	1.943(19)	2.896(3)	170.9(18)
N(15)-H(15C)...N(1)#	0.96(2)	1.88(2)	2.821(3)	165(2)
N(16)-H(16A)...N(9)#	0.91(3)	2.59(3)	3.135(3)	119(3)
N(16)-H(16B)...N(3)#	0.86(3)	2.41(3)	3.262(3)	174(3)
C(5)-H(5C)...N(2)#	0.9600	2.6200	3.433(4)	143.00

Symmetry transformations used to generate equivalent atoms:

#1 x,y,z #2 -x,-y,-z

2. Optimized structures of AHMT cation

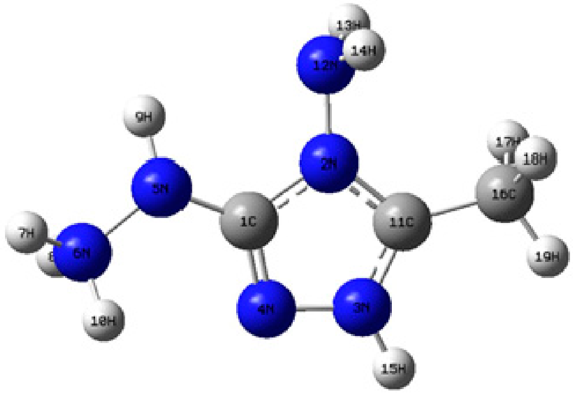

Table S8. Cartesian coordinates of the optimized structure of AHMT cation

	X	Y	Z
C	-0.000001864	-0.000015252	0.00000594
N	0.000040735	0.000018144	0.000022044
N	-0.000017948	-0.000000612	0.000011517
N	-0.000008248	-0.000002821	-0.000035589
N	0.000026855	0.000034864	0.000001986
N	0.00000228	-0.000017613	0.000002945
H	-0.000002098	0.000006048	-0.000000773
H	-0.000006294	0.000002705	0.000000106

H	-0.000006494	-0.00001291	-0.000004597
C	-0.00000311	-0.00000234	0.000009429
N	-0.000014687	-0.000003379	-0.000019379
H	-0.000019593	-0.000005512	-0.000005989
H	0.000003791	-0.00000904	-0.000003273
H	0.000009406	0.00000378	0.000000526
H	-0.000001338	-0.000002017	0.000006369
C	0.000011753	0.000008293	0.000013759
H	-0.000007904	-0.000002094	-0.000003144
H	-0.000003658	-0.000001395	-0.000000236
H	-0.000001584	0.00000115	-0.000001642

3. Heat of formation calculations of AHMT based salts

Based on a Born–Haber energy cycle (Scheme 1), heats of formation of ionic salts can be simplified by Equation:[1-5]

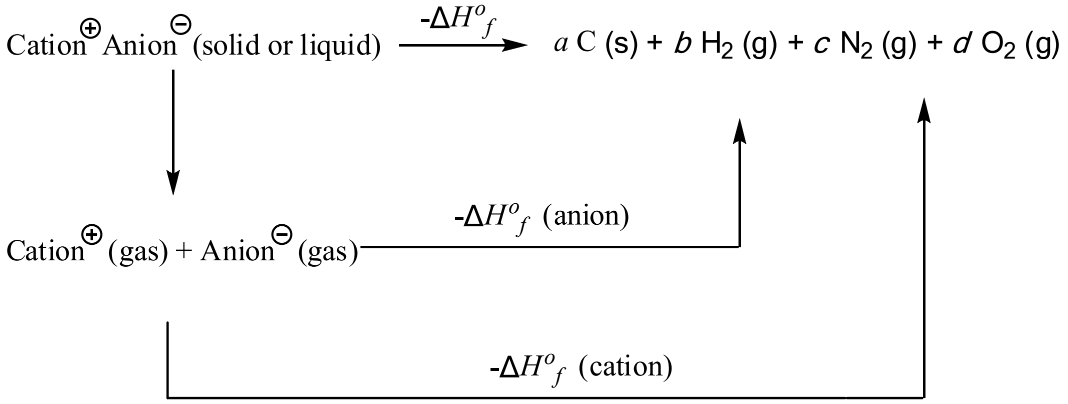

Scheme S1. Born–Haber cycle for the formation for energetic salts.

$$\Delta H_f^o\ (\text{ionic salts, 298K}) = \Sigma\Delta H_f^o\ (\text{cation, 298 K}) + \Sigma\Delta H_f^o\ (\text{anion, 298 K}) - \Delta H_L \qquad (1)$$

Where $\triangle H^\circ_f$ is the lattice energy of the ionic salts, the $\triangle H^\circ_f$ value could be predicted by the formula suggested by Jenkins et al.[6]

$$\Delta H_L = U_{\text{POT}} + [p(nM/2-2) + q(n_{\text{X}}/2-2)]RT \qquad (2)$$

Where UPOT is the lattice potential energy and nM and nX depend on the nature of the ions Mp^+ and Xq^- , respectively, and are equal to three for monoatomic ions, five for linear polyatomic ions, and six for nonlinear polyatomic ions. The equation for lattice potential energy, U_{POT},

takes the form of Eq. (3):

$$U_{\mathrm{POT}}\ (\mathrm{kJ\ mol^{-1}}) = \gamma(\rho_{\mathrm{m}} / M_{\mathrm{m}})^{1/3} + \delta \qquad (3)$$

Where ρ_{m} is density (g·cm^{-3}), M_{m} is the chemical formula mass of the ionic material (g), and the coefficients γ (kJ·mol^{-1}·cm) and δ (kJ·mol^{-1}) are assigned literature values. The heats of formation of the cations were computed by using iso-desmic reactions. The heats of formation of the anion and the parent ions in the isodesmic reactions were calculated from protonation reactions ($\triangle H^{\circ}_f$(H^+) = 1528 kJ·mol^{-1}).[7-9]

Table S9. Calculated total energy (E_0), zero-point energy (ZPE), thermal correction (H_T), and heat of formation (HOF) of AHMT cation.

Compd.	E_0/a.u.	ZPE/(kJ•mol^{-1})	H_T/(kJ•mol^{-1})	HOF/(kJ•mol^{-1})
HTZ^{2+}	-408.8416907	361.59	22.77	2033.5
$AHMT^{2+}$	-448.1857217	433.38	27.61	1978.23
NH3	-56.2141405	90.24	10.02	-46.1[10]
CH4	-40.52615693	117.59	10.01	-74.4[10]
ATZ	-297.5624401	199.39	15.40	318[11]
N2H4	-111.209464	138.13	10.25	95.4[10]
CH3NH2	-95.86368616	168.58	11.39	-22.5[10]
CH3CH3	-79.8416794	195.84	11.62	-84.8[10]
H^+	0	0	6.22	1528[12]

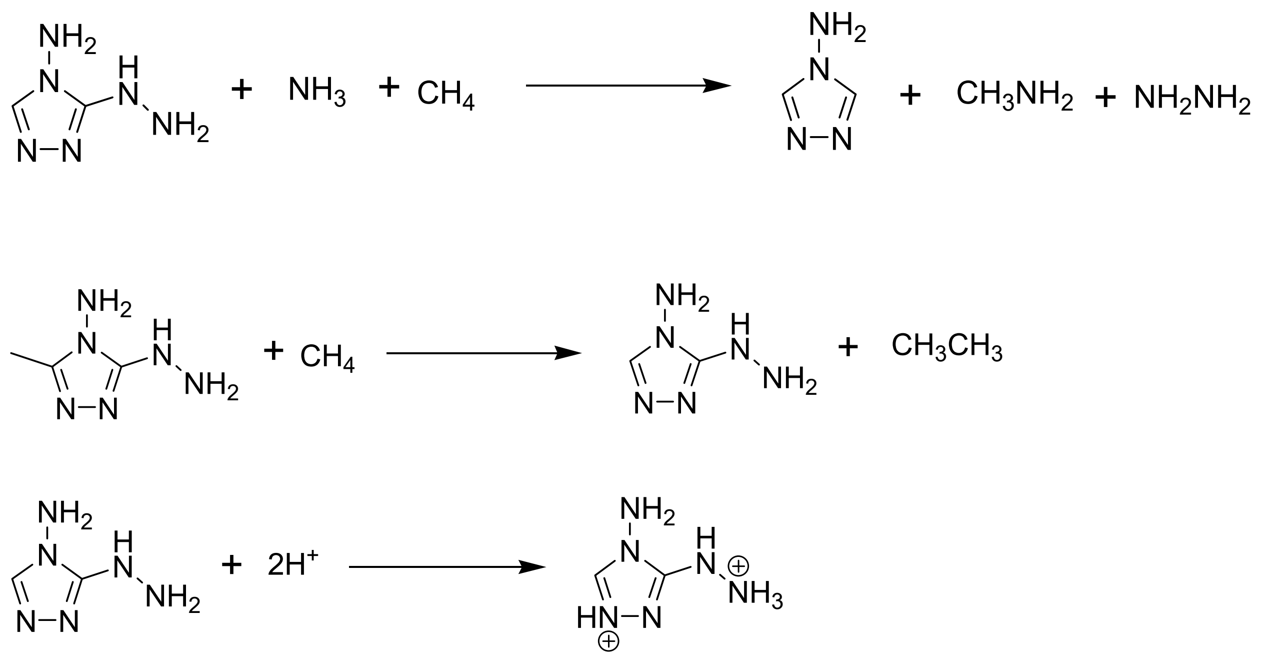

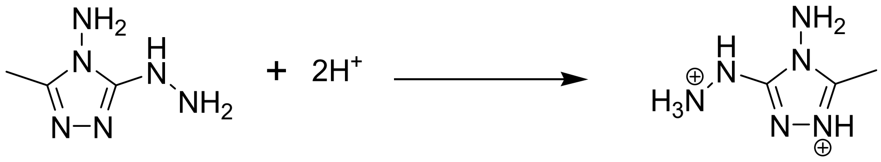

Scheme S2. Isodesmic reactions for **AHT**, **AHMT** and their cations**.**

4. Method of calculated densities

For an ionic crystal with formula unit MpXq, its volume is simply the sum of the volumes of the ions contained in the formula unit: [13-17]

$$V = pV_{M+} + qV_{X-} \qquad (4)$$

where M denotes the cation and X denotes the anion. Because the volumes of individual ions are able to be evaluated using the DFT procedure, we used eq 1 to calculate formula unit volumes for ionic crystals. For those compounds that contain hydrogen atoms, a "corrected" molecular volume using a molecular structure optimized at the DFT level can be calculated using: [13]

$$V(\text{corrected})\text{Opt} = V(\text{uncorrected})\text{Opt} - [0.6763 + 0.9418 \times (\text{no. of hydrogen atoms in the ion})] \qquad (5)$$

Rice et al.[10] reported that the formula unit volumes calculated using the optimized geometries at the B3LYP/6-31G** level and corrected for the number of hydrogen atoms produce average and rms deviations from experimental values of 1.3% and 5.0%, respectively, in much better agreement than the uncorrected values (5.6% and 7.3%, respectively). Therefore, we used the B3LYP/6-31G** method to calculate the molecular volumes for the energetic tetrazolium salts studied here. The volume of each ion was defined as inside a contour of 0.001 electrons bohr^{-3} density that was evaluated using a Monte Carlo integration. We performed 100 single-point calculations for the optimized structure of each ion to get an average volume. For the salts, the theoretical density was obtained from the molecular weight divided by the average molecular volume. This method has been successfully applied to high-nitrogen compounds.[13, 17]

5. Sensitivity

The impact sensitivity was tested on a type 12 tooling according to "up and down" method. A 2.5 kg weight was dropped from a set height onto a 20 mg sample placed on 150 grit garnet sandpaper. Each subsequent test was made at the next lower height if explosion occurred and at the next higher height if no explosion happened. 50 drops were made from different heights, and an explosion or non-explosion was recorded to determine the results. An initial height was made by experiences of the testers based on the structure of the tested compound; several trials at different heights, like 40 cm, 60 cm, 50 cm, 55 cm, 60 cm, were done. For example, when

tested compound **16**, the weight is 5.0 kg and the initial height was set at 55 cm finally since explosion occurred when the height is 63.8 cm, while it did not happen at 40 cm. After that, each subsequent test was made at the next lower height if explosion occurred and at the next higher height if no explosion happened. The test height was spaced at log 0.06 intervals. 50 drops were made from different heights based on the method mentioned above, and an explosion or non-explosion was recorded.

6. General methods

All materials were commercially available and used as received. Melting point was determined using XT4 microscope melting point apparatus. IR spectra were recorded using KBr pellets for solids on a Thermo Nicolet iS10 spectrometer. ^{1}H and ^{13}C spectra were recorded using a 500 MHz (Bruker AVANCE Ⅲ 500) nuclear magnetic resonance spectrometer operating at 500 and 125.72 MHz, respectively. Chemical shifts in the ^{1}H and ^{13}C spectra are reported relative to Me_4Si as external standards. ^{1}H and ^{13}C NMR chemical shifts are reported in ppm relative to TMS. DSC measurements were performed on a differential scanning calorimeter (Mettler Toledo DSC823e) at a scan rate of 5 ℃ min^{-1} in closed Al containers with a nitrogen flow of 50 mL min^{-1}, and calibrated by using indium. Measurements were carried out by heating from 50 °C. Density were measured by using Automatic Density Analyzer, ULTRAPYC 1200. Elemental analyses were carried out using an a vario EL Ⅲ CHNOS elemental analyzer.

7. X-ray Crystallographic Studies

A colorless prism (**3**) of dimensions 0.23 x 0.20 x 0.08 mm, a colorless prism (**5**) of dimensions 0.22 x 0.20 x 0.11 mm, a colorless prism (**9**) of dimensions 0.25 x 0.22 x 0.14 mm were mounted on Rigaku RAXIS RAPID IP diffractometer equipped with a graphite-monochromatized MoKα radiation (λ= 0.71073 Å). An Oxford Cobra low temperature device was used to maintain the crystals at a constant 153(2) K during data collection. Data were collected by ω scan technique. The crystal structure was determined by Rigaku RAXIS IP diffractometer and SHELXTL crystallographic software package of molecular structure. The structure was solved by direct methods with SHELXS-97 and expanded by using the Fourier technique. The non-hydrogen atoms were refined anisotropically. The hydrogen atom was determined with theoretical calculations and refined with an isotropic vibration factor.

CCDC-1516715 (**3**) , 2281718 (**5**) and CCDC-1516716 (**9**) contain the supplementary crystallographic data for this paper. These data can be obtained free of charge from The Cambridge Crystallographic Data Centre via www.ccdc.cam.ac.uk/data_request/cif.

8. Theoretical study

Detonation pressure and velocity were calculated using EXPLO5 v6.01.[18] Computations were performed by using the Gaussian 09 (Revision D.01) suites of programs.[19] We used the B3LYP/6-31G** method to calculate the molecular volumes for the energetic triazolium salts.[20] The volume of each ion was defined as inside a contour of 0.001 electrons/$bohr^3$ density that was evaluated using a Monte Carlo integration. The geometric optimization and the frequency analyses were carried out using B3LYP functional analyses with the 6-311+G** basis set. Single energy points were calculated at the MP2/6-311++G** level. All of the

optimized structures were characterized to be true local energy minima on the potential energy surface without imaginary frequencies.

9. [1]H Spectra of AHMT salts.

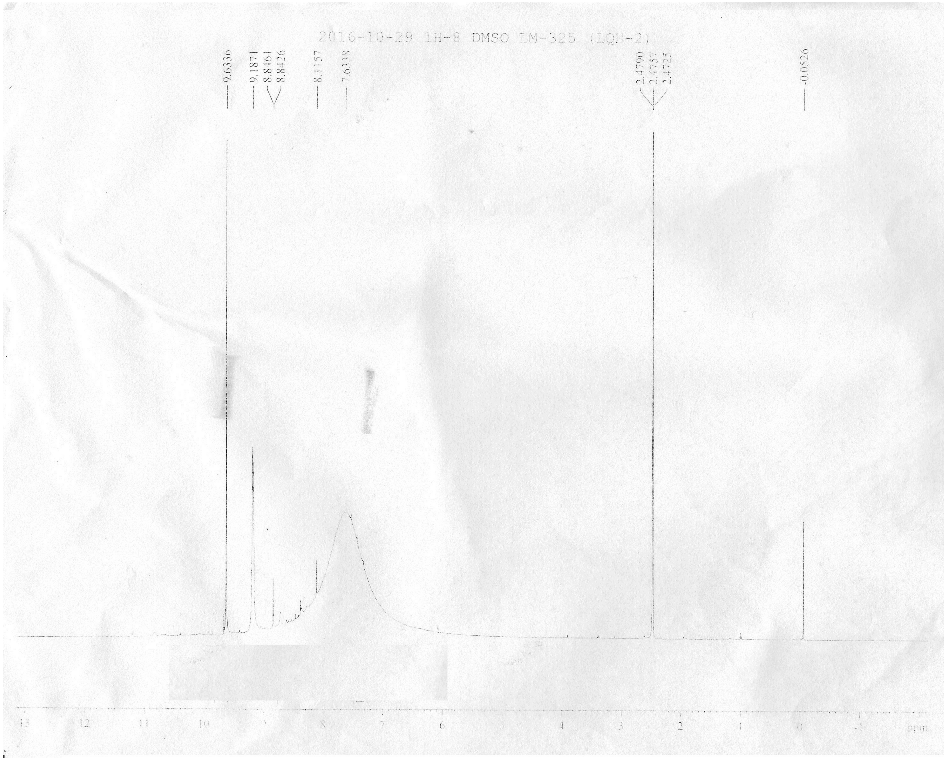

Figure S1. ^{1}H spectra of **3** from DMSO.

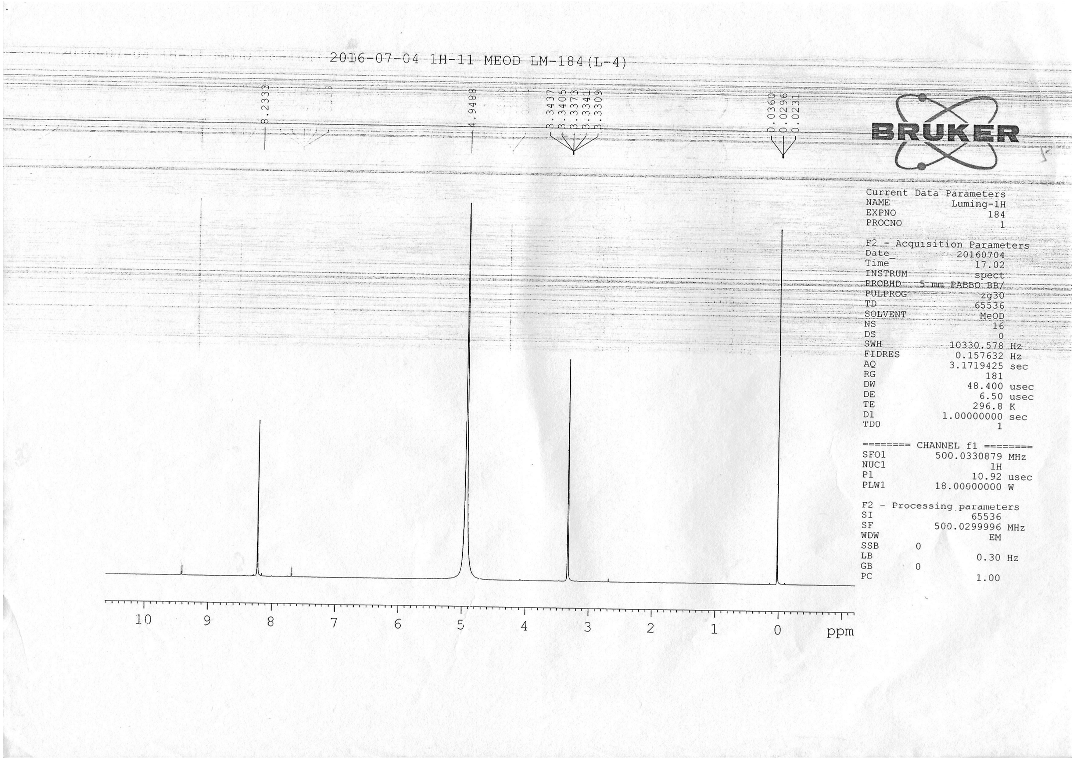

Figure S2. ^{1}H spectra of **5** from MeOD.

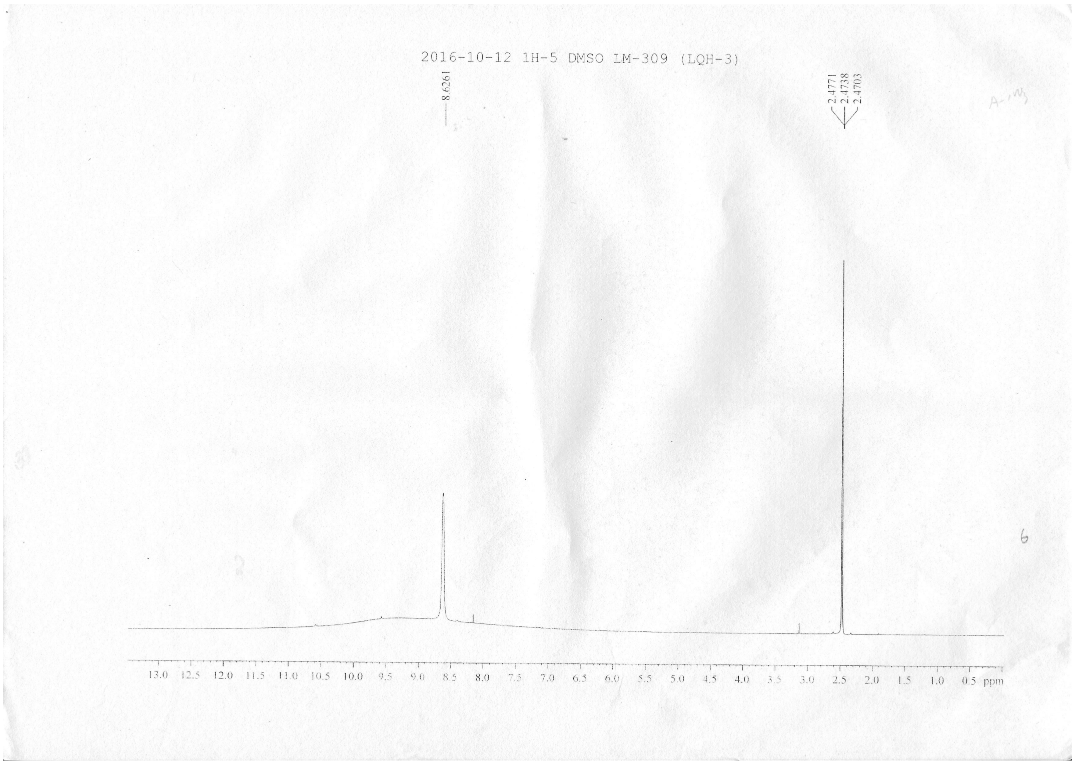

Figure S3. ^{1}H spectra of **6** from DMSO.

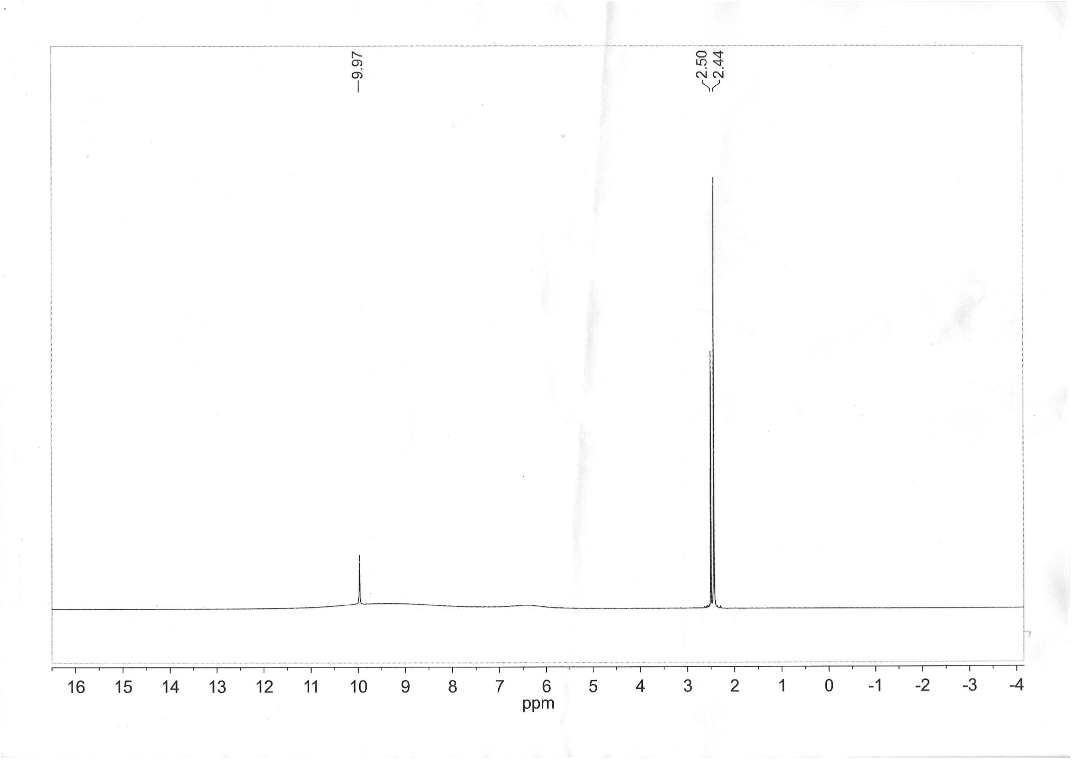

Figure S4. ^{1}H spectra of **7** from DMSO.

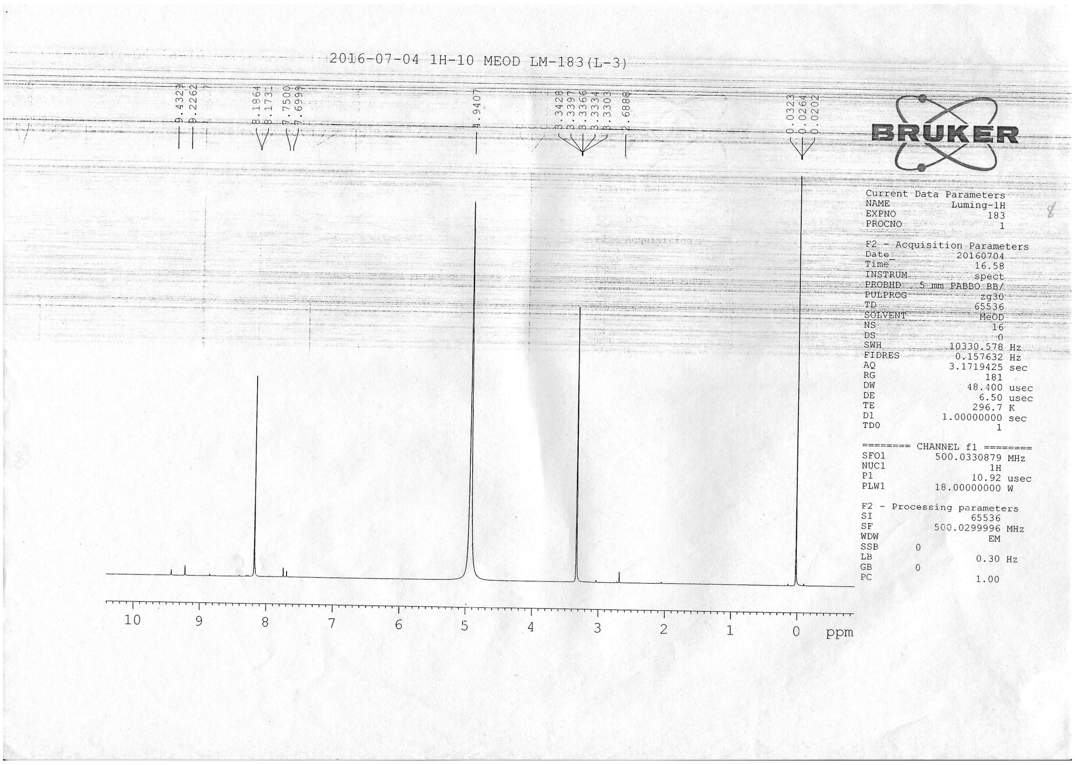

Figure S5. ^{1}H spectra of **8** from MeOD.

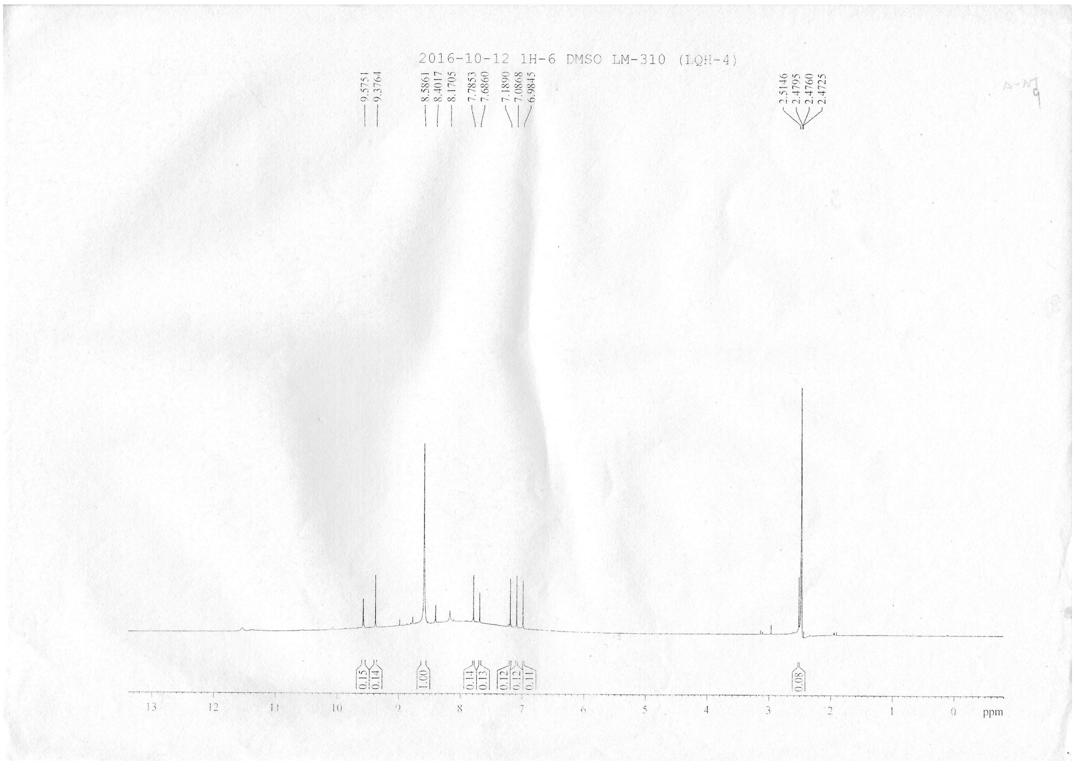

Figure S6. ^{1}H spectra of **9** from DMSO.

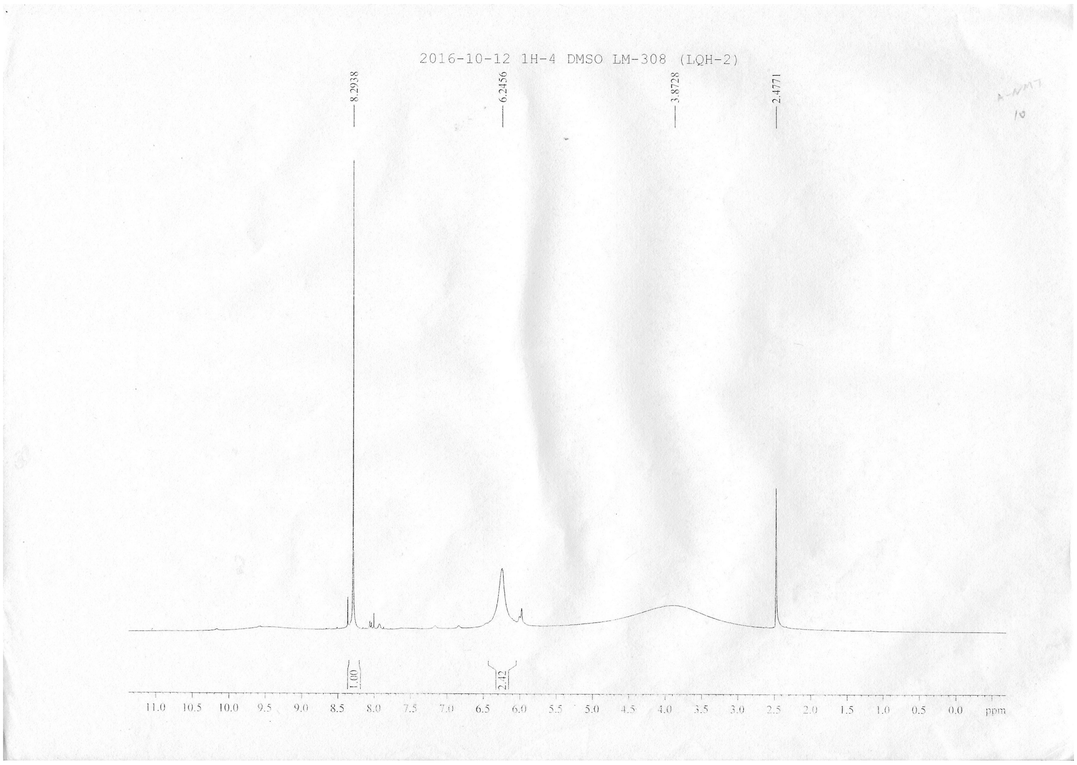

Figure S7. ^{1}H spectra of **10** from DMSO.

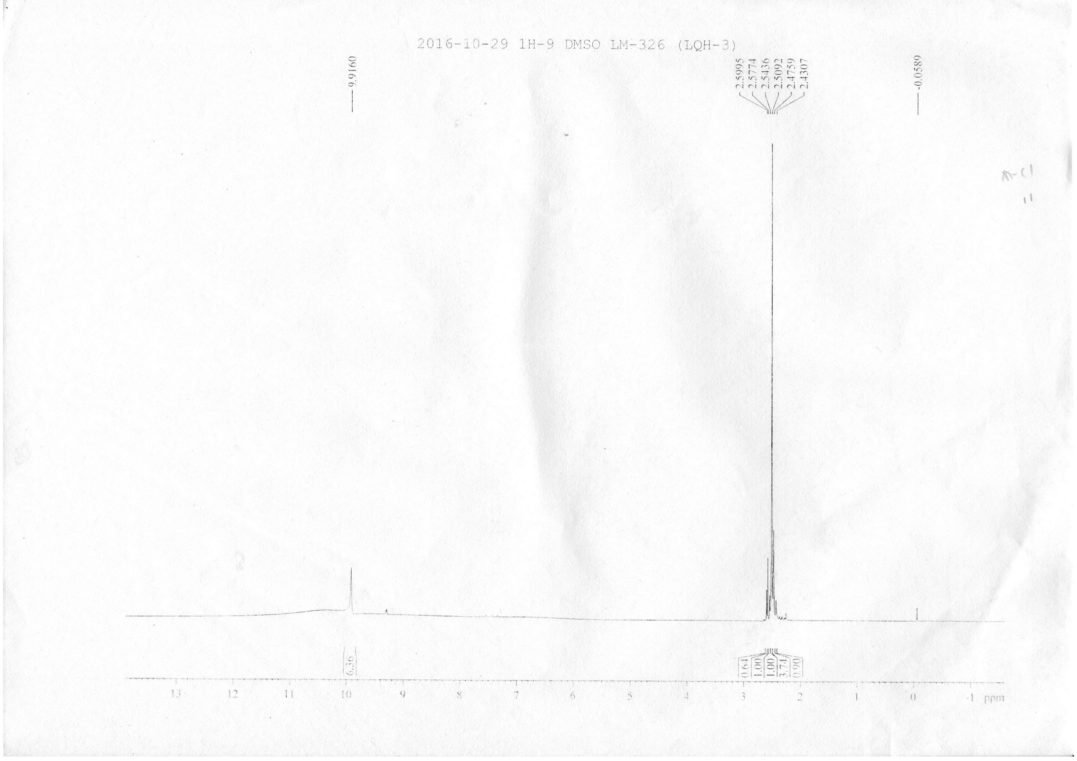

Figure S8. ^{1}H spectra of **11** from DMSO.

10. ^{13}C Spectra of AHMT salts.

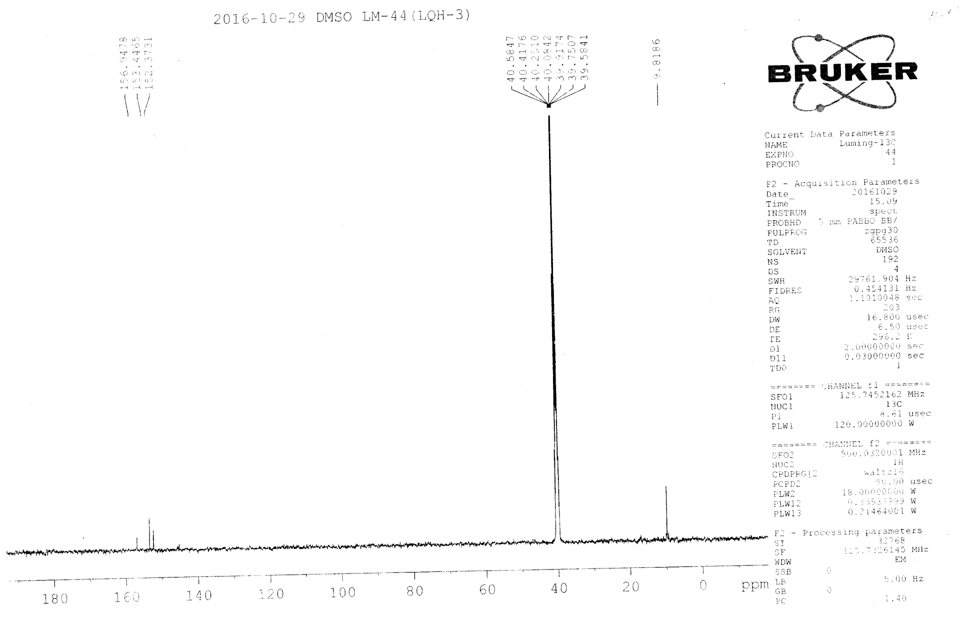

Figure S9. ^{13}C spectra of **3** from DMSO.

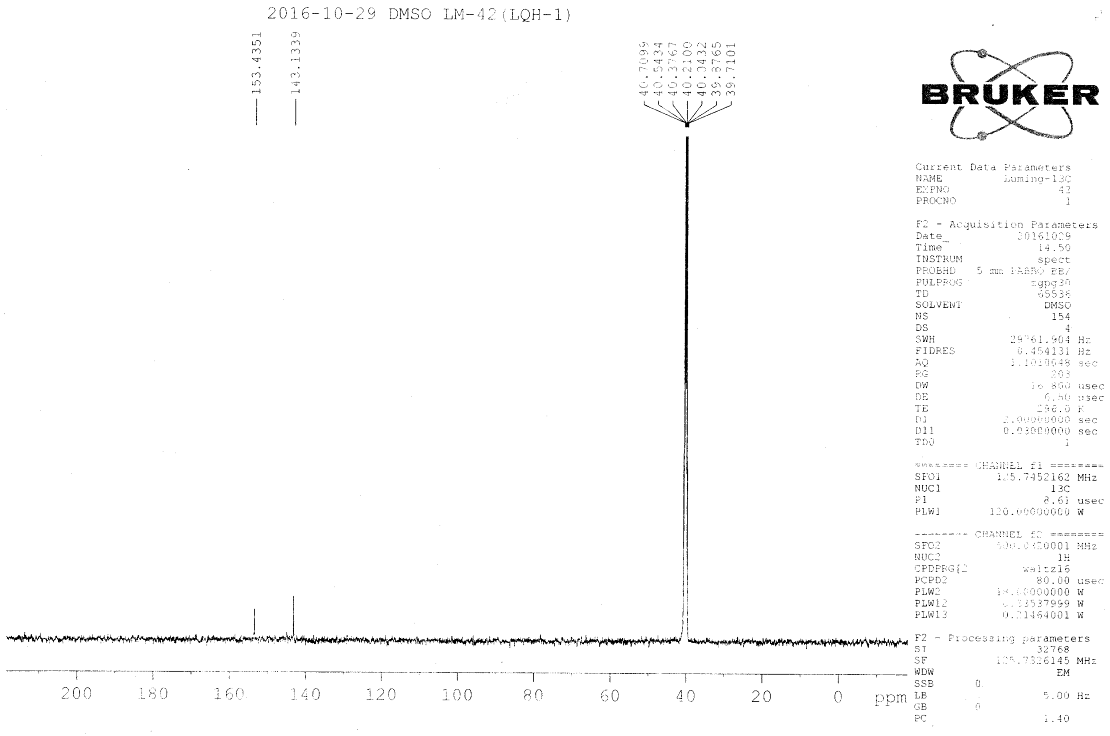

Figure S10. ^{13}C spectra of **4** from DMSO.

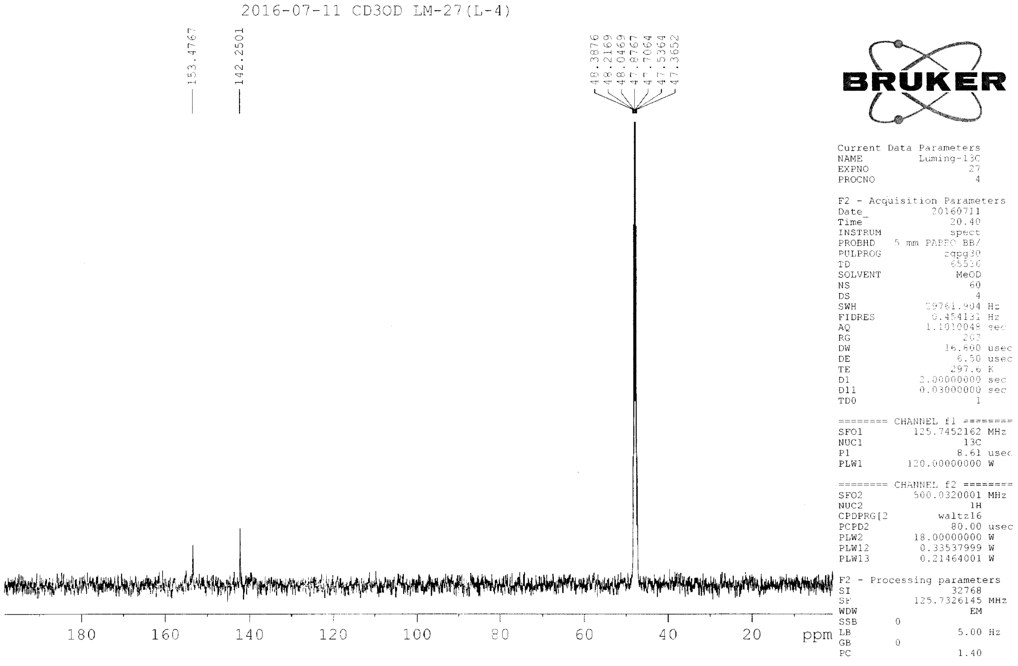

Figure S11. ^{13}C spectra of **5** from CD4O.

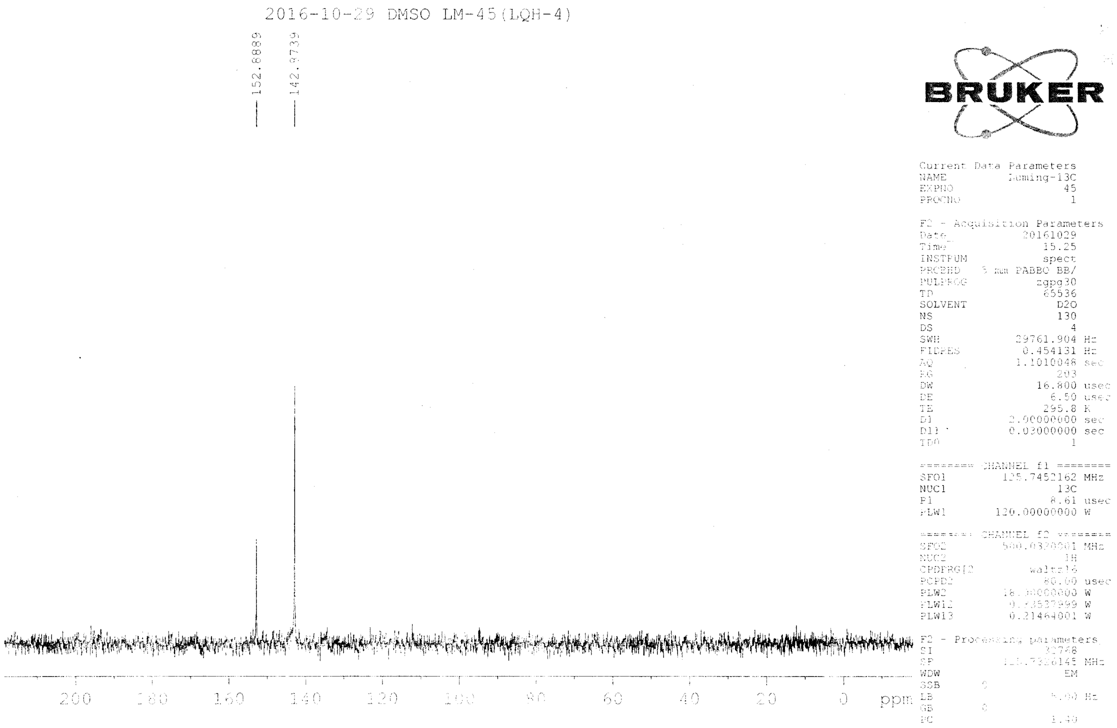

Figure S12. ^{13}C spectra of **7** from DMSO.

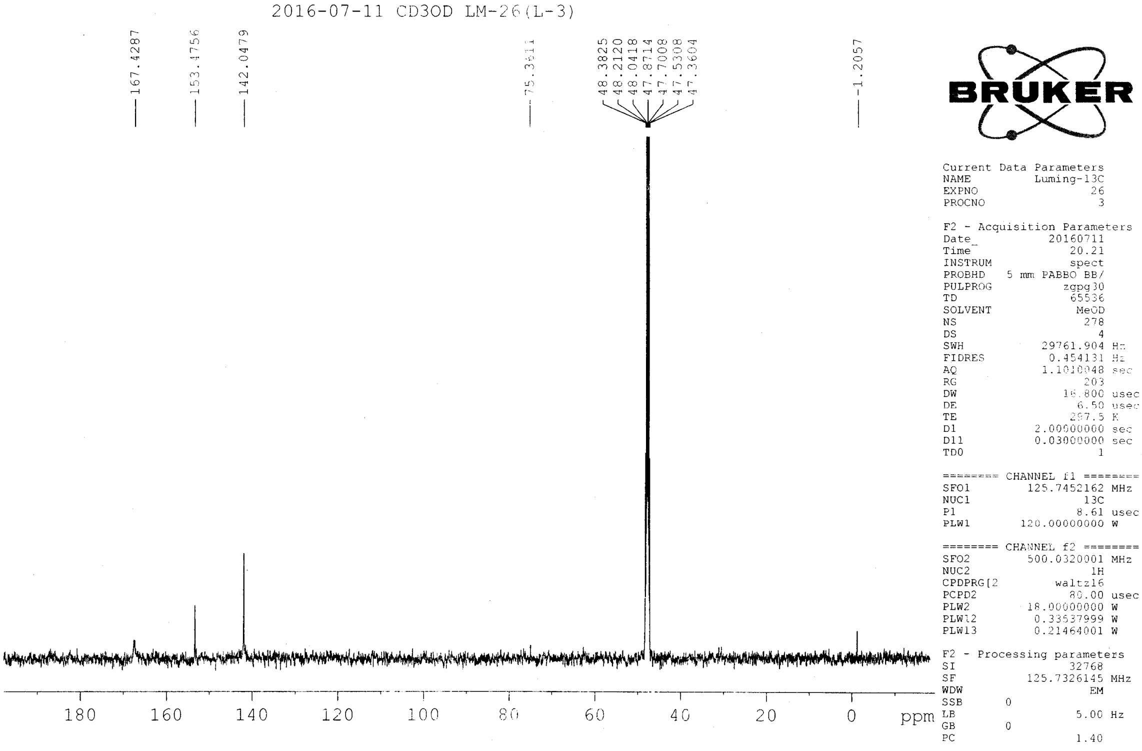

Figure S13. ^{13}C spectra of **8** from CD4O.

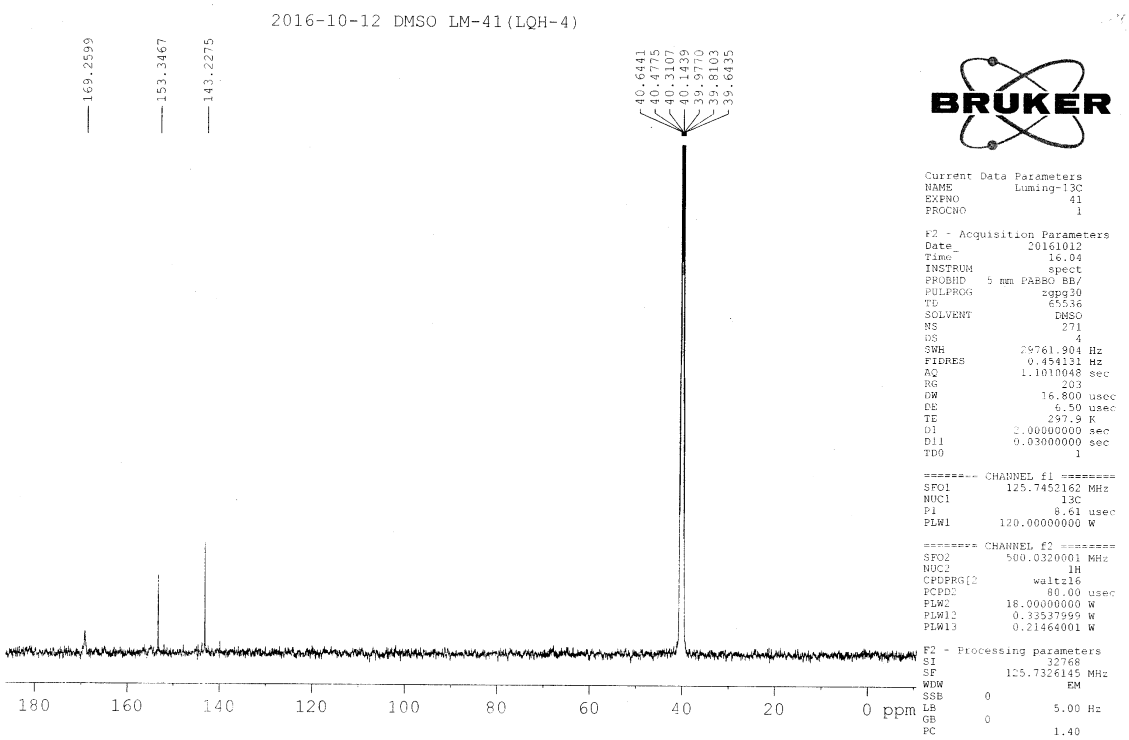

Figure S14. ^{13}C spectra of **9** from DMSO.

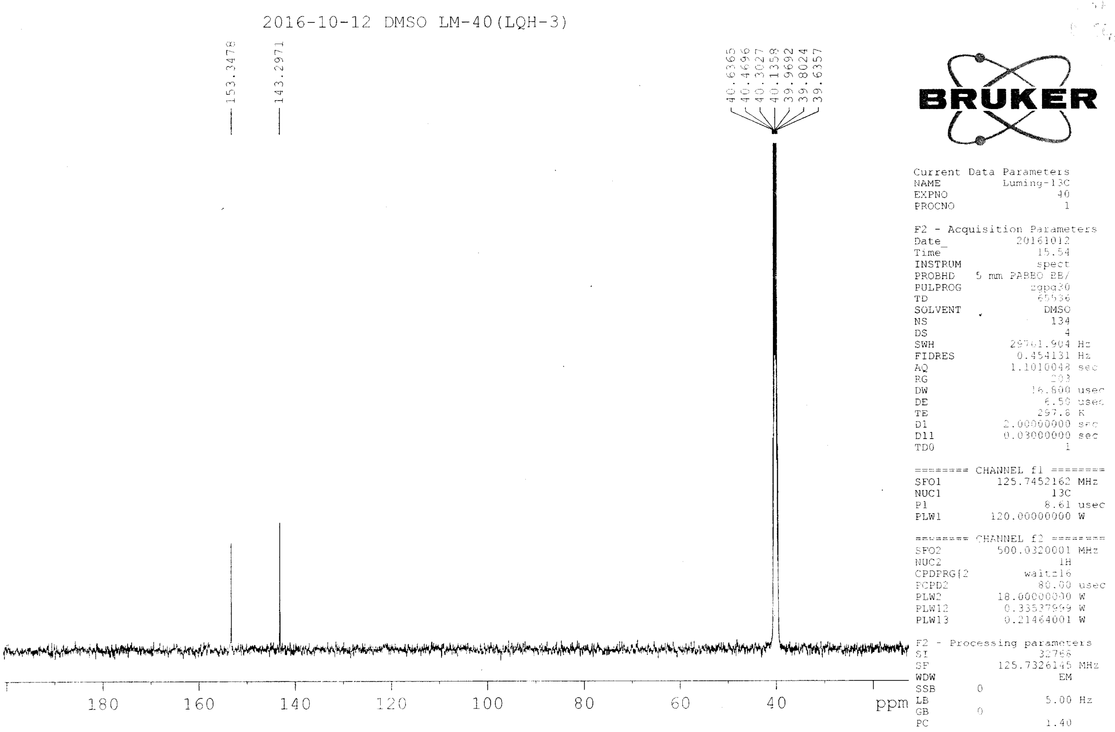

Figure S15. ^{13}C spectra of **10** from DMSO.

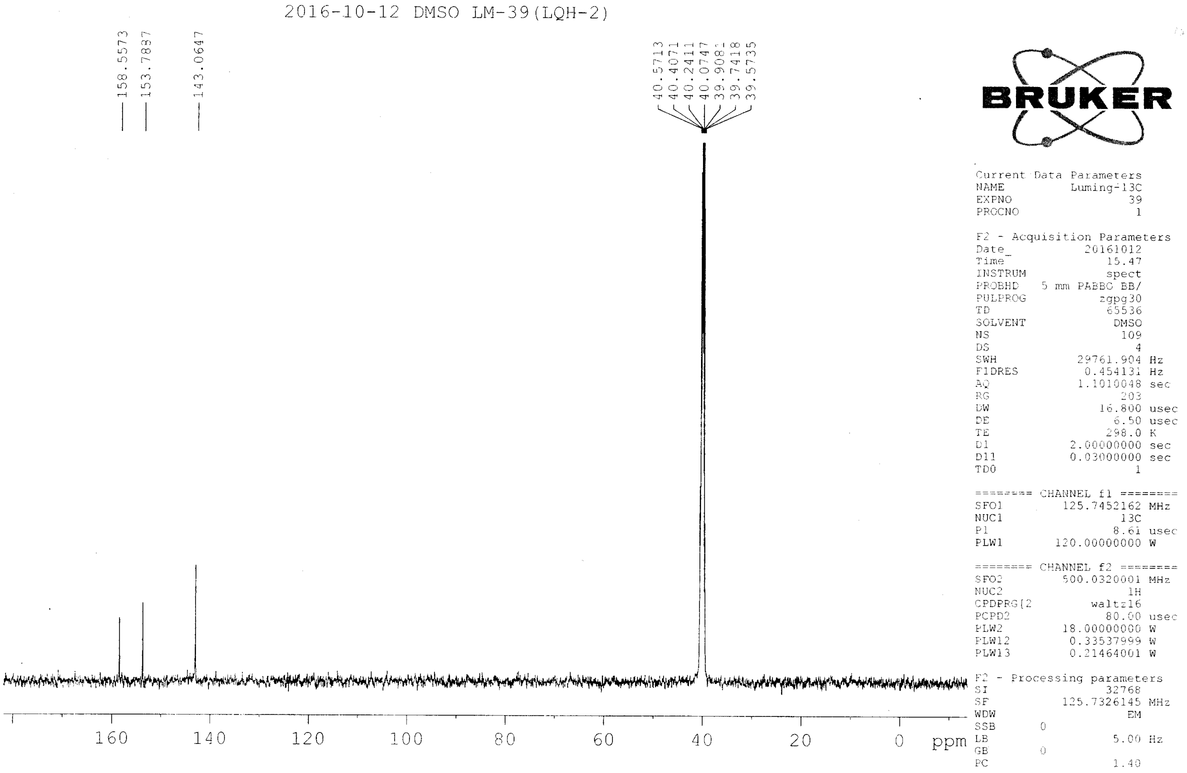

Figure S16. ^{13}C spectra of **11** from DMSO.

11. IR Spectra of AHMT salts.

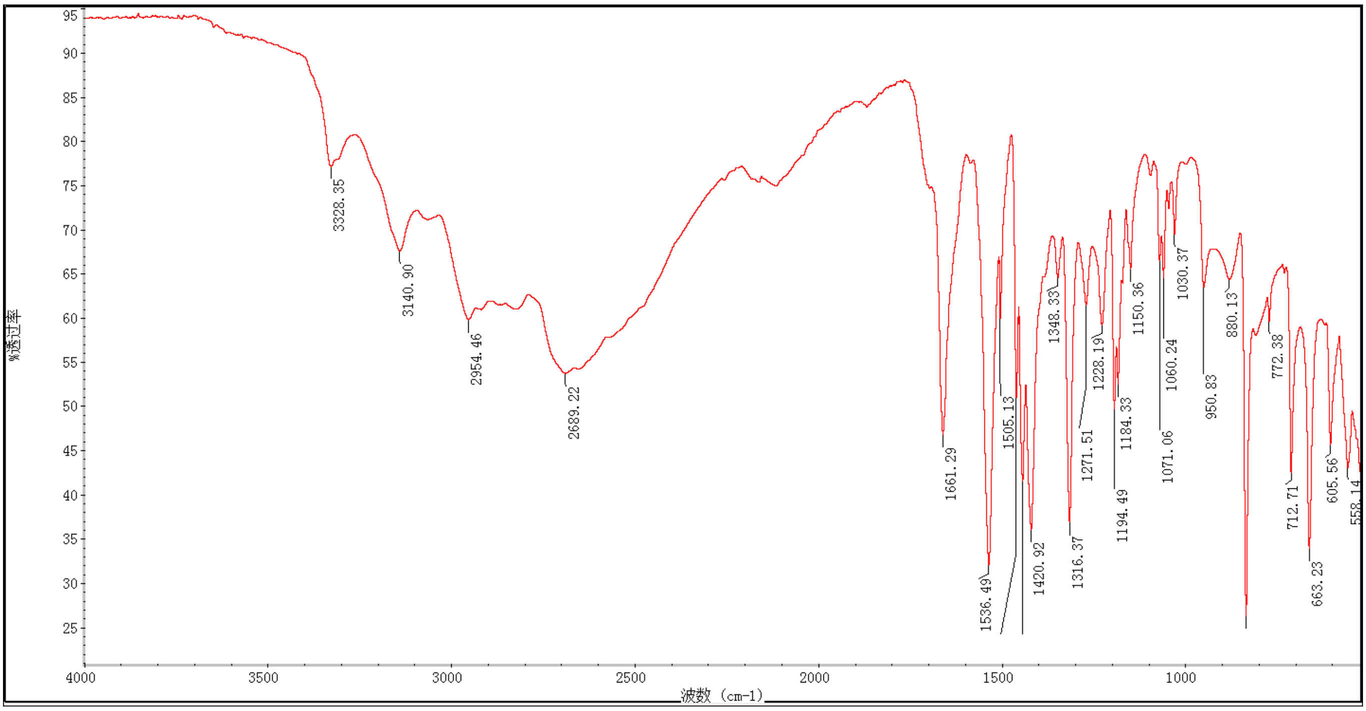

Figure S17. IR spectra of **3**.

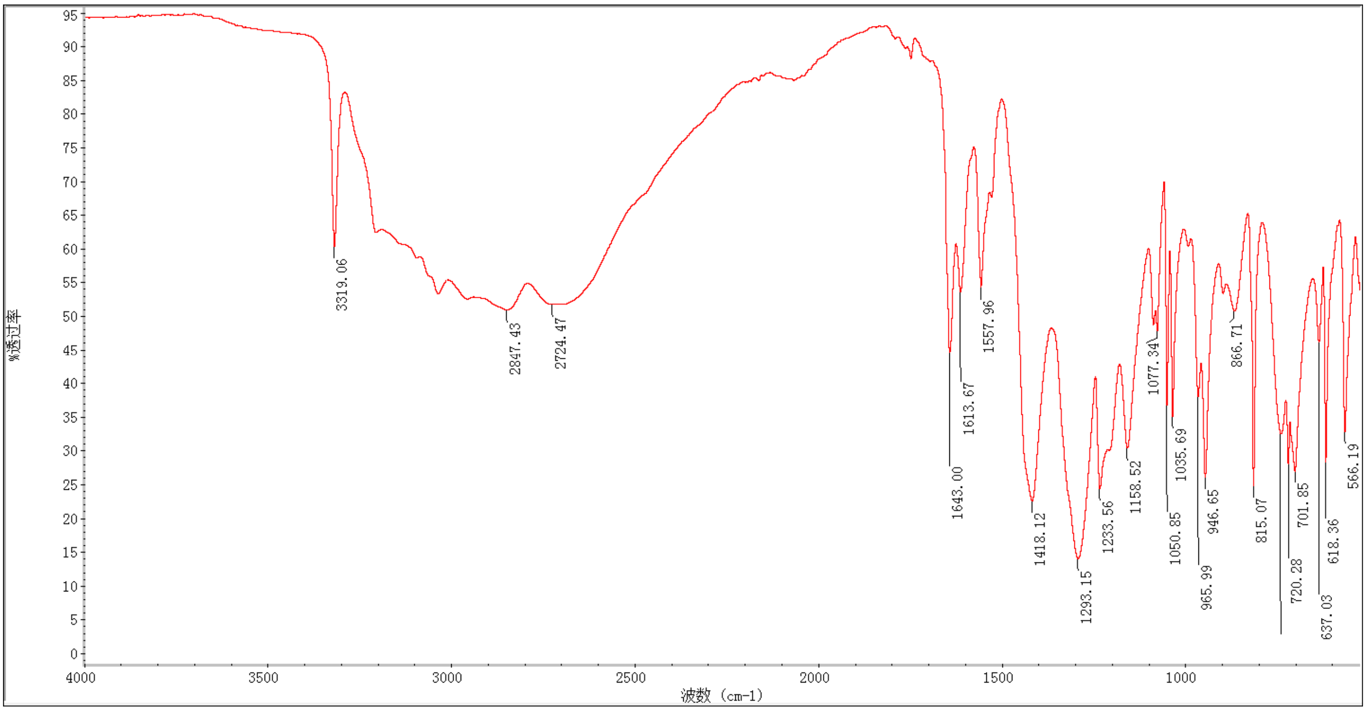

Figure S18. IR spectra of **5**.

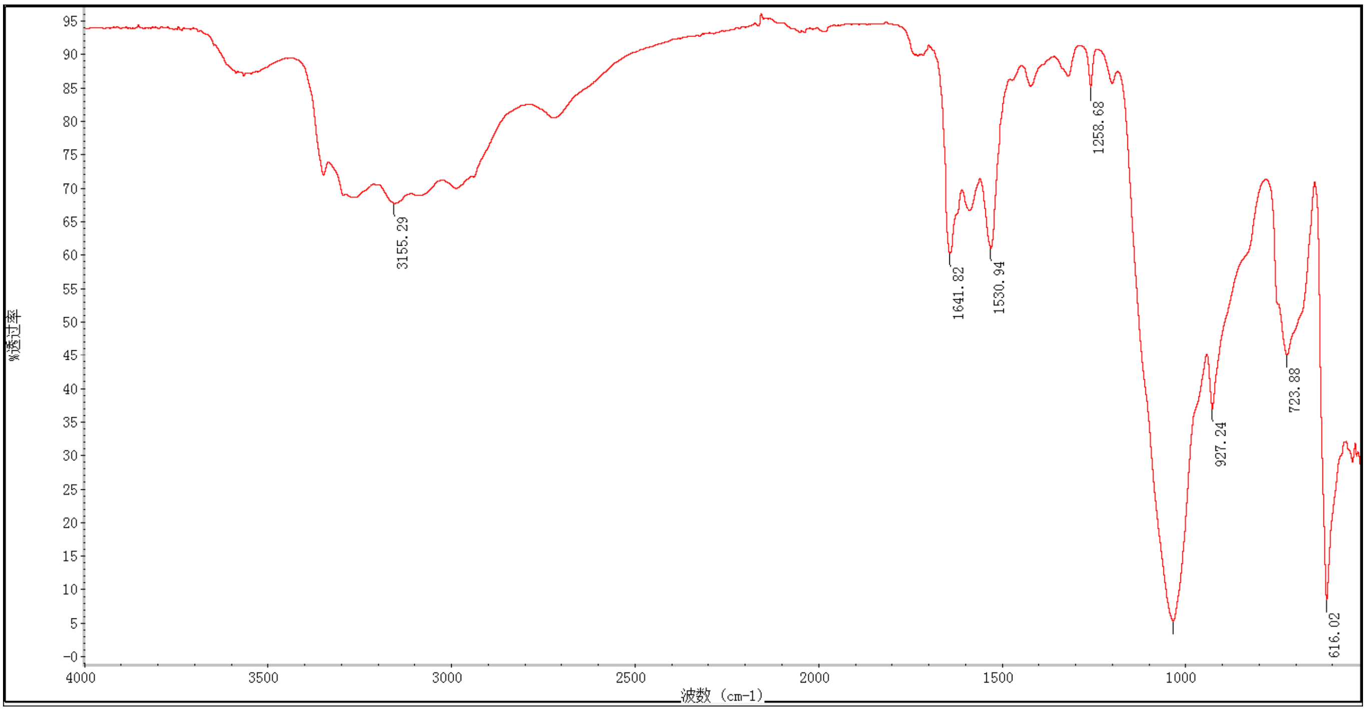

Figure S19. IR spectra of **6**.

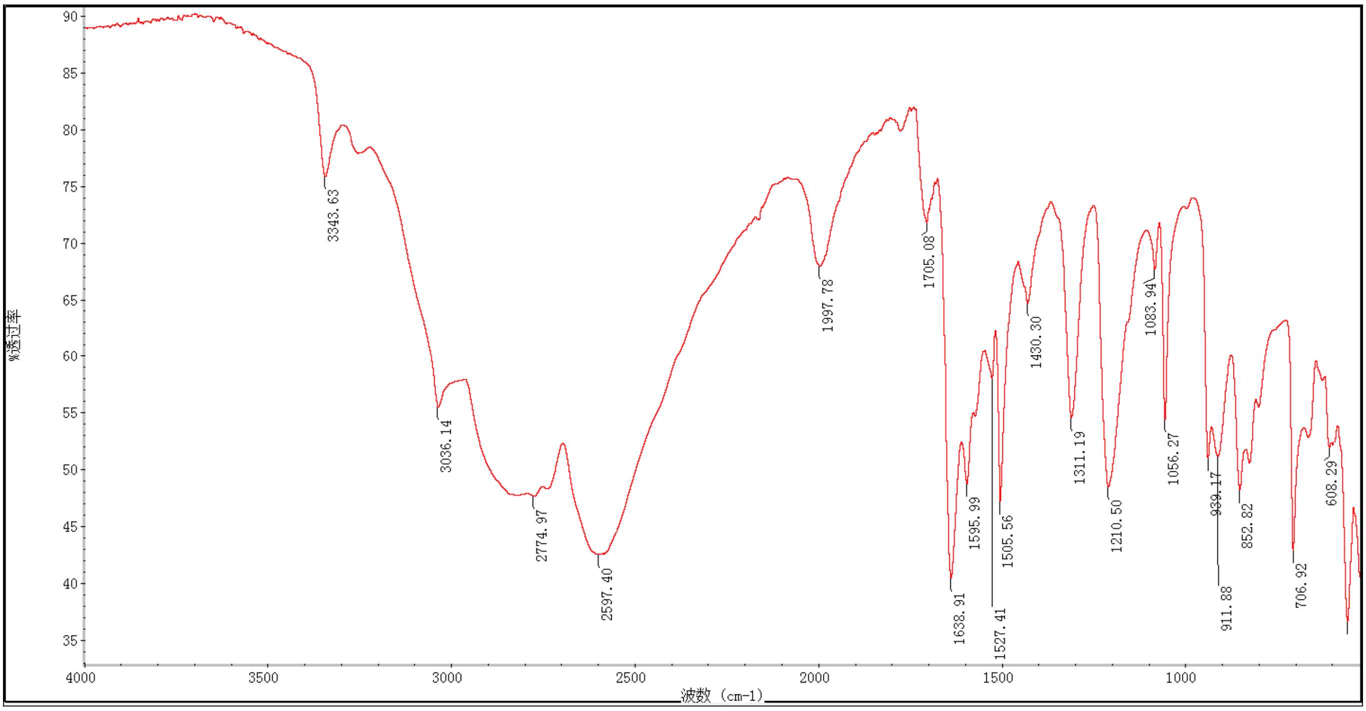

Figure S20. IR spectra of **8**.

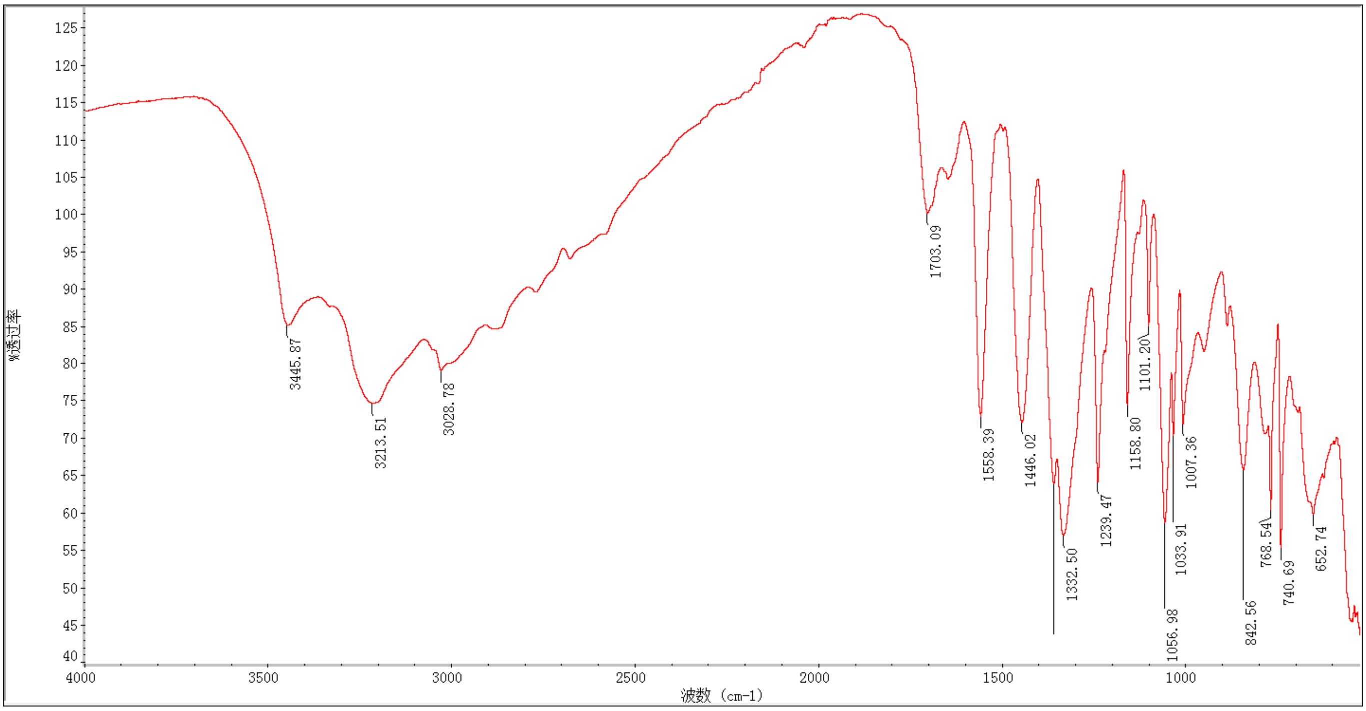

Figure S21. IR spectra of **10**.

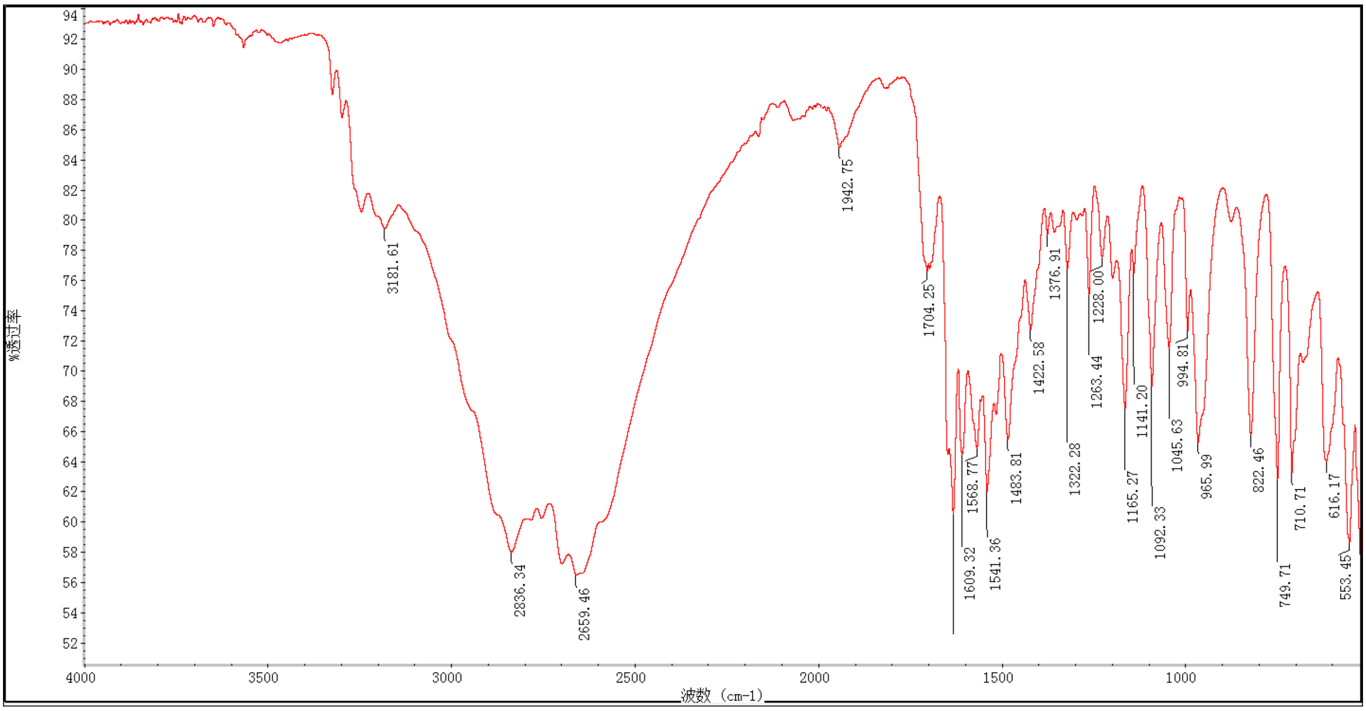

Figure S22. IR spectra of **11**.

12. DSC thermogram of the related compounds

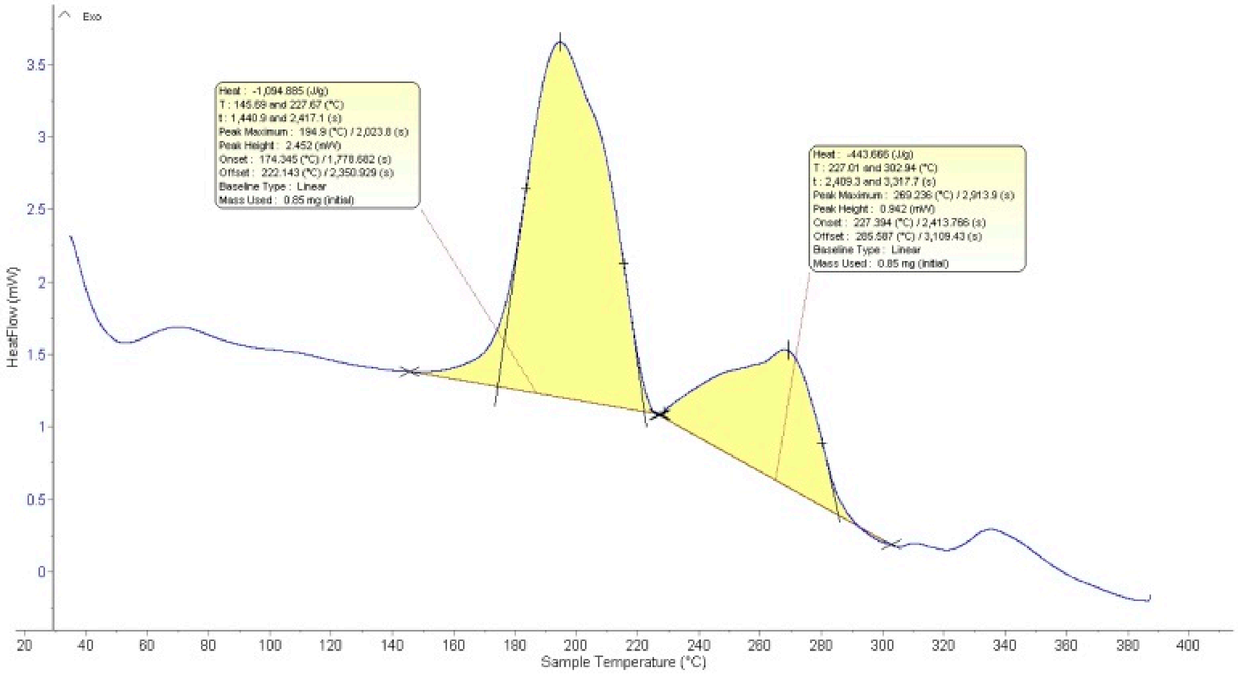

Figure S23. DSC thermogram of **4** (heating rate of 5°C·min^{-1}).

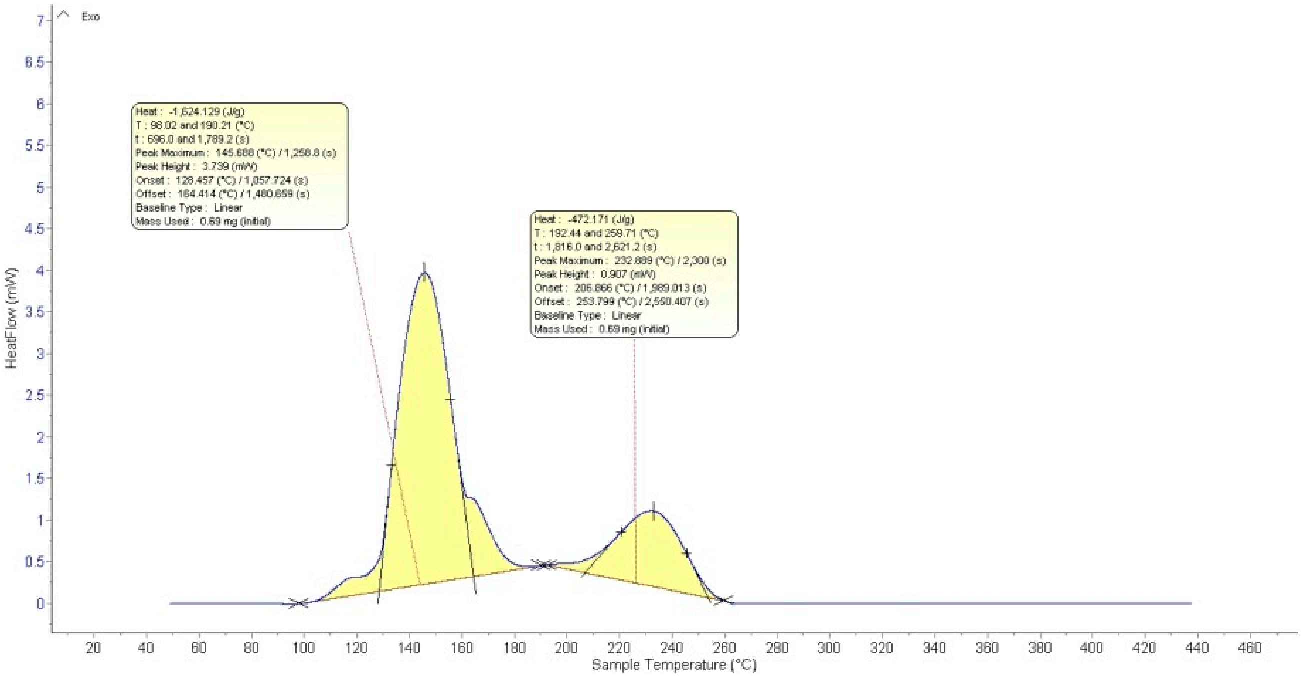

Figure S24. DSC thermogram of **5** (heating rate of 5°C·min^{-1}).

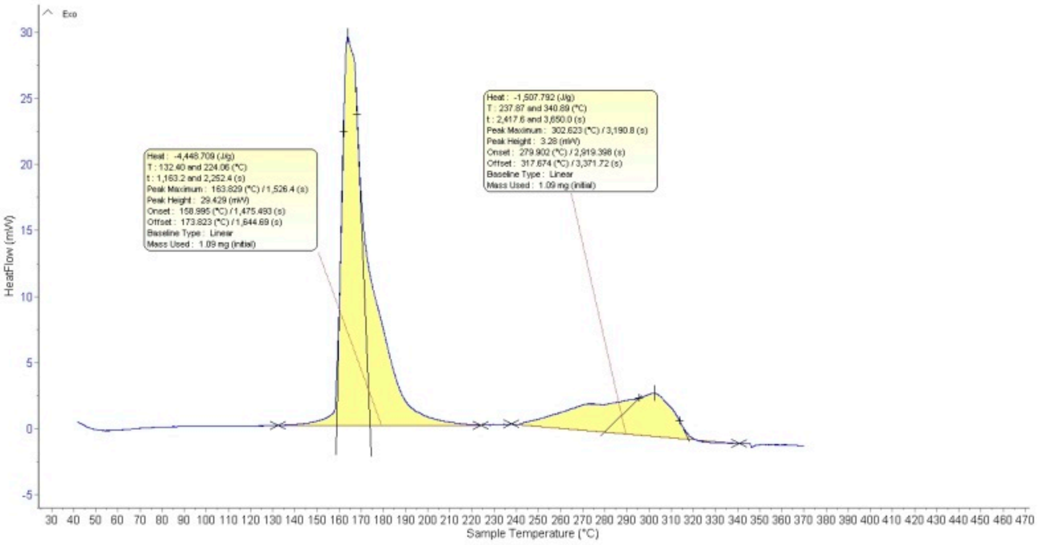

Figure S25. DSC thermogram of **6** (heating rate of 5°C·min^{-1}).

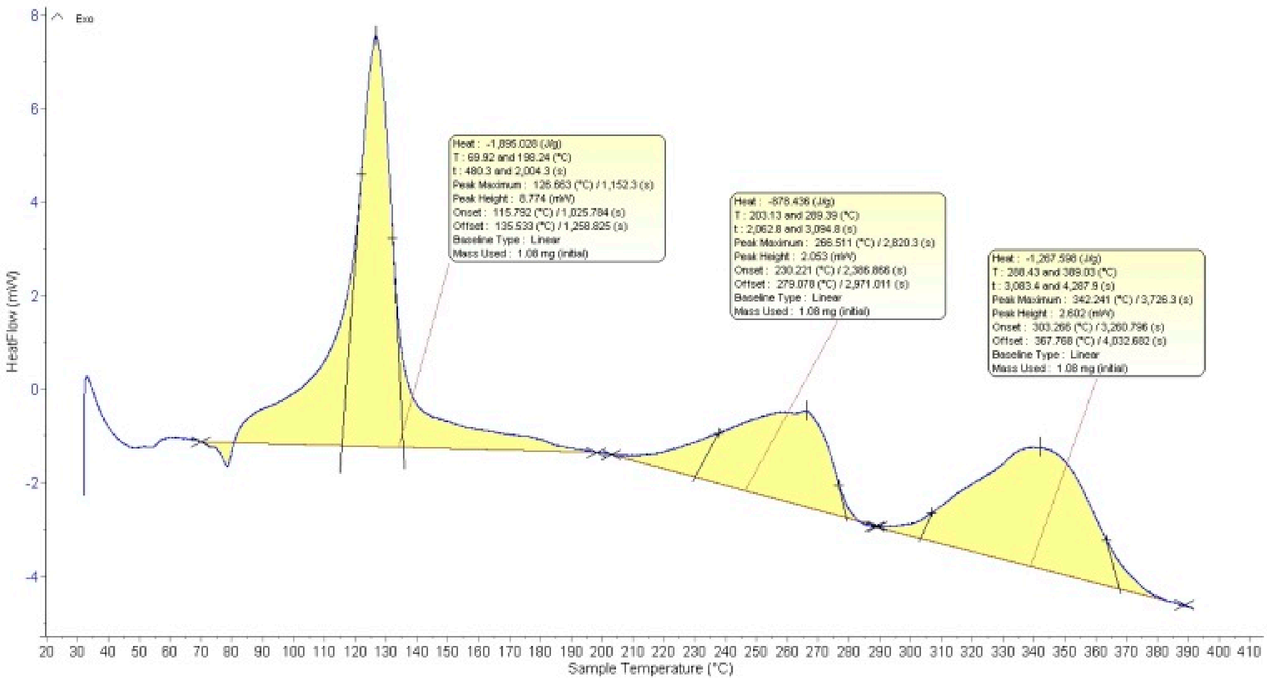

Figure S26. DSC thermogram of **7** (heating rate of 5°C·min^{-1}).

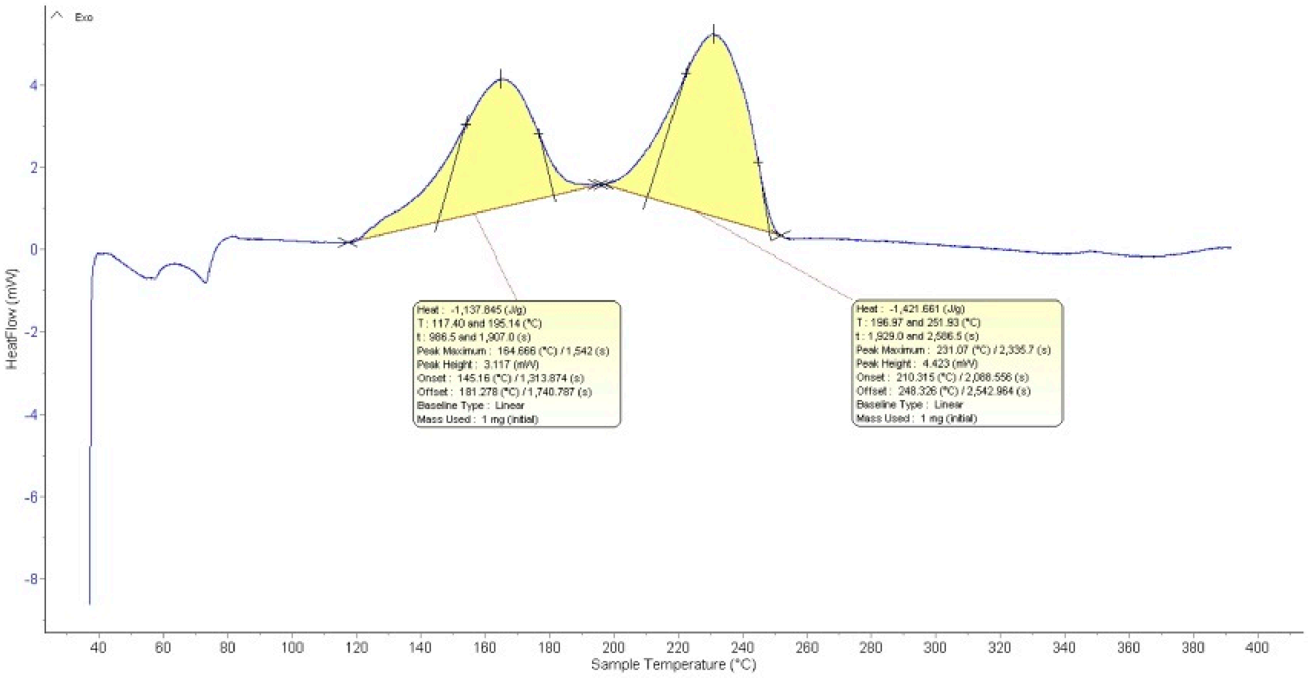

Figure S27. DSC thermogram of **8** (heating rate of 5°C·min^{-1}).

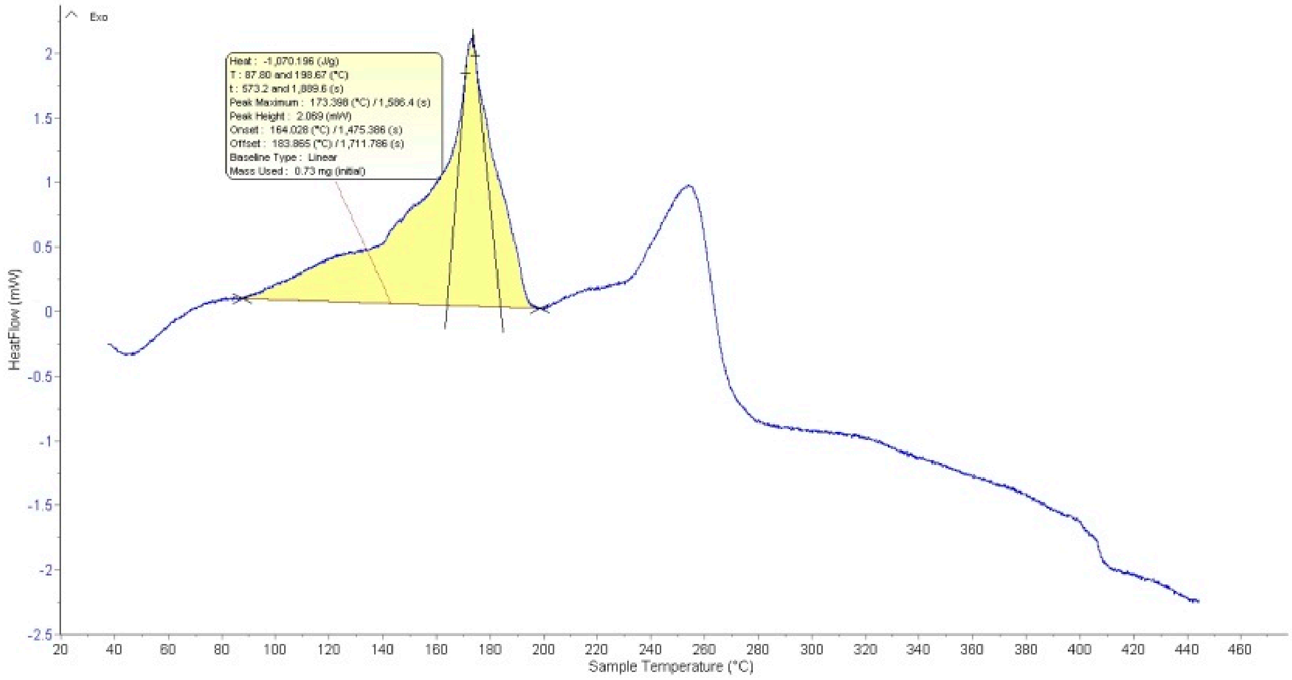

Figure S28. DSC thermogram of **9** (heating rate of 5°C·min^{-1}).

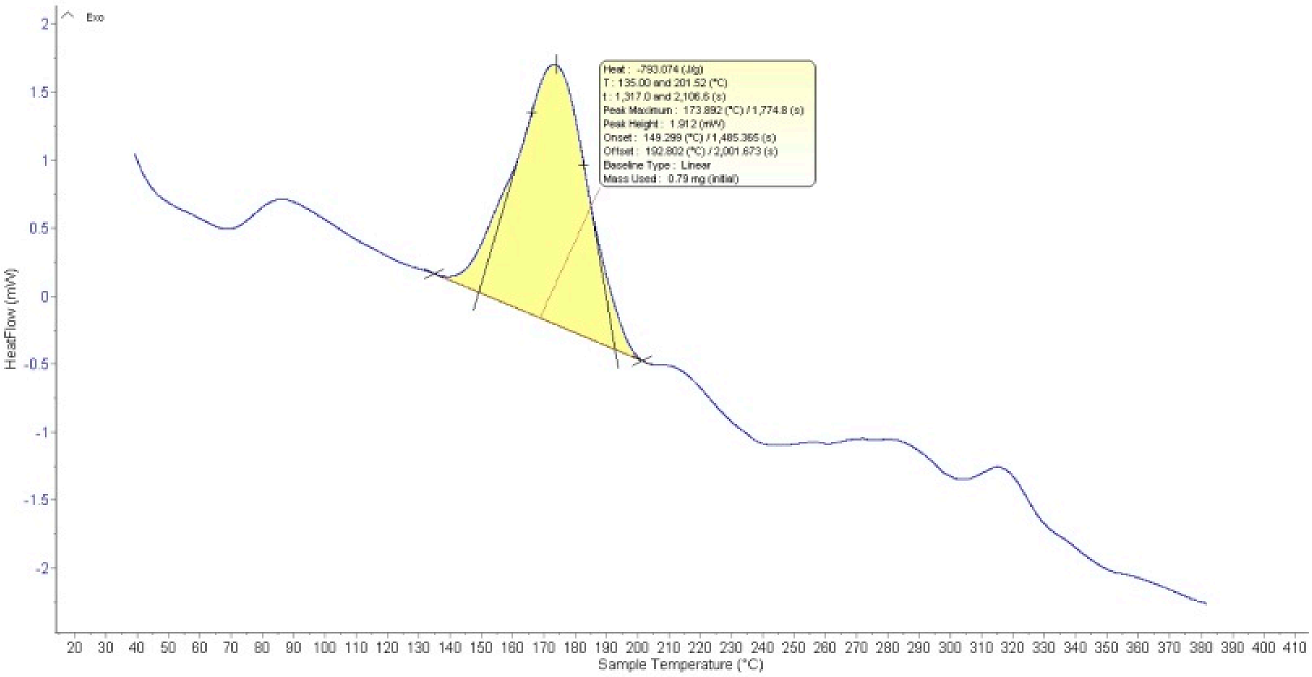

Figure S29. DSC thermogram of **11** (heating rate of 5°C·min^{-1}).

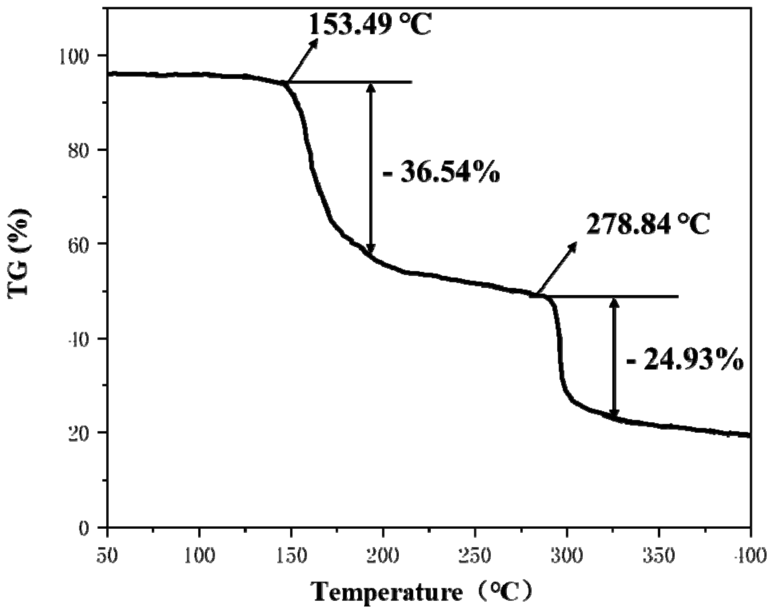

Figure S30. TG thermogram of **6** (heating rate of 5°C·min^{-1}).

13. References

1. Suceska, M. EXPLO5—Computer Program for Calculation of Detonation Parameters. In Proceedings of the 32nd International Annual Conference of ICT, Karlsruhe, Germany, 3–6 July 2001.

2. Frisch, M.J.; Trucks, G.; Schlegel, H.B.; Scuseria, G.E.; Robb; Cheeseman, J.R.; Scalmani, G.; Barone, V.; Mennucci, B.; Petersson, G.A. Gaussian 09W; revision A. 02; Gaussian Inc., Wallingford CT, **2009**.
3. Spackman, M.A.; Jayatilaka, D. Hirshfeld surface analysis. *CrystEngComm* **2009**, *11*, 19-32; DOI:10.1039/B818330A.
4. Spackman, P.R.; Turner, M.J.; McKinnon, J.J.; Wolff, S.K.; Grimwood, D.J.; Jayatilaka, D.; Spackman, M.A. CrystalExplorer: a program for Hirshfeld surface analysis, visualization and quantitative analysis of molecular crystals. *J Appl Crystallogr* **2021**, *54*, 1006-1011; DOI: 10.1107/S1600576721002910.
5. Johnson, E.R.; Keinan, S.; Mori-Sánchez, P.; Contreras-García, J.; Cohen, A.J.; Yang, W. Revealing Noncovalent Interactions. *J. Am. Chem. Soc.* **2010**, *132*, 6498-6506; DOI: 10.1021/ja100936w.
6. Lu, T.; Chen, F. Multiwfn: A multifunctional wavefunction analyzer. *J. Comput., and Chem* **2012**, *33*, 580-592; DOI: 10.1002/jcc.22885.
7. Humphrey, W.; Dalke, A.; Schulten, K. VMD: Visual molecular dynamics. *J MOL GRAPH MODEL* **1996**, *14*, 33-38; DOI: 10.1016/0263-7855(96)00018-5.
8. Zhang, J.; Lu, T. Efficient evaluation of electrostatic potential with computerized optimized code. *Phys. Chem. Chem. Phys* **2021**, *23*, 20323-20328; DOI: 10.1039/D1CP02805G.
